# A Robust Finite Element Method for Linearized Magnetohydrodynamics on General Domains

**DOI:** 10.1007/s10915-026-03291-y

**Published:** 2026-04-11

**Authors:** L. Beirão da Veiga, C. Lovadina, M. Trezzi

**Affiliations:** 1 Dipartimento di Matematica e Applicazioni, Università degli Studi di Milano Bicocca, Via Roberto Cozzi 55, 20125 Milano, Italy; 2 IMATI-CNR, Via Adolfo Ferrata 5, 27100 Pavia, Italy; 3 Dipartimento di Matematica “F. Enriques”, Università degli Studi di Milano, Via Cesare Saldini 50, 20133 Milano, Italy

**Keywords:** MHD, Finite element, Pressure Robust

## Abstract

We generalize and improve the finite element method for linearized Magnetohydrodynamics introduced in (Beirão da Veiga et al., SIAM J. Numer. Anal. **62**(4):1539–1564 (2024)). The main novelty is that the proposed scheme is able to handle also non-convex domains and less regular solutions. The method is proved to be pressure robust and quasi-robust with respect to both fluid and magnetic Reynolds numbers. A set of numerical tests confirms our theoretical findings.

## Introduction

Recently, the field of magnetohydrodynamics (MHD) has garnered increasing attention within the computational mathematics community. MHD equations are relevant in the study of plasmas and liquid metals, finding applications across geophysics, astrophysics, and engineering. The combination of fluid dynamics and electromagnetism equations results in a variety of models. These models have different formulations and lead to several finite element choices, each with its own advantages and disadvantages. Examples of relevant works in this area include [3, 4, 6, 16, 19–29] (this list is not exhaustive).

In this paper we consider, as in [6], the linearized version of the following three field formulation of the MHD problem:

1 $$\begin{aligned} \left\{ \begin{aligned}&\text { find } (\boldsymbol{u}, p, \boldsymbol{B}) \text { such that}\\&\begin{aligned} \boldsymbol{u}_t - \nu _\textrm{S}\, \boldsymbol{\textrm{div}}(\boldsymbol{\varepsilon }(\boldsymbol{u}) ) + (\boldsymbol{\nabla }\boldsymbol{u}) \,\boldsymbol{u}+ \boldsymbol{B}\times \boldsymbol{\textrm{curl}}(\boldsymbol{B}) - \nabla p&= \boldsymbol{f}\qquad  &   \text {in } \Omega \times I, \\ \textrm{div}\, \boldsymbol{u}&= 0 \qquad  &   \text {in } \Omega \times I, \\ \boldsymbol{B}_t + \nu _\textrm{M}\, \boldsymbol{\textrm{curl}}(\boldsymbol{\textrm{curl}}(\boldsymbol{B}) ) - \boldsymbol{\textrm{curl}}(\boldsymbol{u}\times \boldsymbol{B})&= \boldsymbol{G}\qquad  &   \text {in } \Omega \times I, \\ \textrm{div}\, \boldsymbol{B}&= 0 \qquad  &   \text {in } \Omega \times I. \end{aligned} \end{aligned} \right. \end{aligned}$$

Above, $\Omega$ is a spatial three-dimensional domain, while *I* is a time interval. Moreover, $\boldsymbol{u}$, *p* and $\boldsymbol{B}$ are the velocity, pressure and magnetic fields, respectively; the parameters $\nu _S$ an $\nu _M$ represent the fluid and the magnetic diffusion coefficients, respectively. We recall that in several engineering and physical applications, the scaled parameters $\nu _S$ and, potentially, $\nu _M$ are quite small. For instance, in aluminum electrolysis, $\nu _S$ is approximately 1e-5 and $\nu _M$ is around 1e-1 (see, for example, [2, 8]). Even smaller values of $\nu _M$ are encountered, for instance, in geophysics and space weather prediction problems. When these parameters are small, it is well-known that standard finite elements become unstable and stabilization techniques are necessary to achieve reliable numerical results. In the literature there are only a few finite element schemes that are (provably) robust for small values of $\nu _S,\nu _M$, such as [6, 19, 24, 29] for the linear case and, to our knowledge, only [7] for the fully nonlinear case.

The presented scheme takes the initial steps from the method in [6] but, contrary to such contribution which is focused to convex domains $\Omega$, we here allow for general domains, possibly non-convex. Indeed, the method in [6] makes use of an $H^1$-conforming discrete magnetic field, which cannot guarantee convergence for general non-convex domains, as it is well-known and also confirmed by our numerical test in Section 6.

We highlight that the present extension is not merely a straightforward adaptation of the tools used for the convex case. Instead, considering non-convex domains implies deeper changes, including a different framework for the variational formulation of Problem (1), cf. Remark 2.2 in [6]. The finite element scheme here presented yields the following set of important advantages:

1. pressure robustness, i.e. in particular the fluid velocity error does not depend on the pressure error;
2. quasi-robustness with respect to dominant advection, i.e. assuming a sufficiently regular solution, the estimates are independent of both $\nu _S$ and $\nu _M$ in an error norm that controls convection);
3. suitable also for non-convex domains;
4. suitability for extension to the time-dependent case using a standard time-stepping scheme, unlike SUPG or similar methods.

Note that, to our best knowledge, none of the existing schemes in the literature share all the above properties.

To achieve these objectives also for general domains, rather than the space $\boldsymbol{H}^1$ we first consider a variation formulation involving the space $\boldsymbol{H}(\boldsymbol{\textrm{curl}})$ for the magnetic field, and select the Nédélec finite element of second type for its discretization. As a consequence, the divergence free condition for the magnetic field $\boldsymbol{B}$, see fourth equation of (1), cannot be directly inserted in the variational formulation, but it is recovered by the assumption that $\boldsymbol{G}$ is orthogonal to the gradients.

The fluid velocity and pressure discrete spaces, as well as the formulation employed to ensure robustness with respect to all regimes, are as follows:

- for pressure robustness, velocity is approximated by means of $\boldsymbol{H}(\textrm{div})$-conforming $\boldsymbol{\textrm{BDM}}$ elements, while standard discontinuous piecewise polynomials are used for the pressure discretization. Hence, the velocity approximation space gives rise to a non-conforming scheme, which needs to be stabilized. Stability is then recovered by adding face terms in the spirit of the DG methods;
- in order to be robust with respect to the fluid diffusion parameter, a typical upwinding DG term is included in the variational formulation;
- to deal with the fluid-magnetic coupling, and to be robust also with respect to the magnetic diffusion parameter, suitable Continuous Interior Penalty (CIP) terms are introduced.

Even though we borrow some techniques and results from [6], we highlight that our analysis represents a significant improvement over the one developed in that paper. In particular, it must be observed that the technique adopted in [6] to exploit the CIP (Continuous Interior Penalty) stabilization in the convergence proofs is not suitable for the present setting. The main reason is that such an approach, based on a peculiar orthogonality property, prevents the use of specific Nédélec interpolants, which are instead needed to handle the kind of solutions that are encountered in magnetic problems on non-convex domains. Therefore we take a different route, and prove a stronger stability with respect to a norm associated with the linearization of the convective term $\boldsymbol{\textrm{curl}}(\boldsymbol{u}\times \boldsymbol{B})$. This approach, although initially more involved, has two advantages:

1. it allows more freedom in the choice of the interpolant during the convergence analysis, as commented here above;
2. it shows stability in a stronger norm which also includes a magneto-advective term, thus providing a solid theoretical justification of what it is observed in the numerical experiments, i.e. the scheme robustness in the magneto-advective regime.

In addition, we present some numerical results that clearly show the failure of the method described in [6] (well-suited for convex domains), when applied to a kind of 3D L-shaped domain.

The paper is organized as follows. Section 2 introduces the continuous problem under consideration, i.e. the linearized version of Problem (1), together with a suitable variational formulation, capable to deal with possibly non-convex domains. Section 3 presents notation and preliminary results, useful for the subsequent study. Section 4 details our proposed stabilized scheme, by introducing the necessary discrete spaces and forms. Section 5 develops the theoretical convergence analysis: first, we prove a stability result involving norms that can also handle the advection-dominated regime; secondly, the optimal error estimates in those norms are derived. The numerical tests are presented in Section 6. As already mentioned, in addition to support the theoretical estimates, the numerical experiments give evidence that $\boldsymbol{H}^1$-conforming approximations for the magnetic field are unable to converge when general non-convex domains are involved. Furthermore, an example of application to a Hartmann flow problem is provided.

Finally, throughout the paper we use standard notations for functional (Sobolev) spaces and their norms/seminorms. However, we explicitly recall the definition of the following spaces.

2 $$\begin{aligned} \begin{aligned}&L^2_0(\Omega ):= \left\{ v \in L^2(\Omega ) \,\,\,\text {s.t.} \,\,\, \int _\Omega v = 0 \right\} \ ,\\&\boldsymbol{H}_0(\textrm{div}, \Omega ) := \{\boldsymbol{v}\in \boldsymbol{L}^2(\Omega ) \,\,\,\text {s.t.} \,\,\, \textrm{div}\,\boldsymbol{v}\in L^2(\Omega ) \,\,\, \text {and} \,\,\, \boldsymbol{v}\cdot \boldsymbol{n}= 0 \,\,\, \text {on } \partial \Omega \}\ ,\\&\boldsymbol{H}(\boldsymbol{\textrm{curl}}, \Omega ) := \{\boldsymbol{H}\in \boldsymbol{L}^2(\Omega ) \,\,\,\text {s.t.} \,\,\, \boldsymbol{\textrm{curl}}(\boldsymbol{H}) \in \boldsymbol{L}^2(\Omega ) \}\ ,\\&\boldsymbol{H}^r(\boldsymbol{\textrm{curl}}, \Omega ) := \{\boldsymbol{H}\in \boldsymbol{H}^r(\Omega ) \,\,\,\text {s.t.} \,\,\, \boldsymbol{\textrm{curl}}(\boldsymbol{H}) \in \boldsymbol{H}^r(\Omega ) \} \ \ \ (r> 0)\, . \end{aligned} \end{aligned}$$

## Model Problem

We consider a linearized version of problem (1), which reads as

3 $$\begin{aligned} \left\{ \begin{aligned}&\text { find } (\boldsymbol{u}, p, \boldsymbol{B}) \text { such that}\\&\begin{aligned} \sigma _\textrm{S}\, \boldsymbol{u}- \nu _\textrm{S}\, \boldsymbol{\textrm{div}}(\boldsymbol{\varepsilon }(\boldsymbol{u}) ) + (\boldsymbol{\nabla }\boldsymbol{u}) \,\boldsymbol{\chi }+ \boldsymbol{\Theta }\times \boldsymbol{\textrm{curl}}(\boldsymbol{B}) - \nabla p&= \boldsymbol{f}\qquad  &   \text {in } \Omega , \\ \textrm{div}\, \boldsymbol{u}&= 0 \qquad  &   \text {in } \Omega , \\ \sigma _\textrm{M}\, \boldsymbol{B}+ \nu _\textrm{M}\, \boldsymbol{\textrm{curl}}(\boldsymbol{\textrm{curl}}(\boldsymbol{B}) ) - \boldsymbol{\textrm{curl}}(\boldsymbol{u}\times \boldsymbol{\Theta })&= \boldsymbol{G}\qquad  &   \text {in } \Omega , \\ \textrm{div}\, \boldsymbol{B}&= 0 \qquad  &   \text {in } \Omega , \end{aligned} \end{aligned} \right. \end{aligned}$$

coupled with the homogeneous boundary conditions

4 $$\begin{aligned} \boldsymbol{u}= 0 \,, \quad \boldsymbol{B}\cdot \boldsymbol{n}= 0 \,, \quad \boldsymbol{\textrm{curl}}(\boldsymbol{B}) \times \boldsymbol{n}= 0 \quad \text {on }\partial \Omega \, . \end{aligned}$$

Here above we denote by $\sigma _\textrm{S}$ and $\sigma _\textrm{M}$ the reaction coefficients (constant for simplicity), and with $\boldsymbol{\Theta }, \boldsymbol{\chi }$ the advective fields of the fluid and the magnetic field, respectively. For the time being, we assume $\boldsymbol{\Theta }\in \boldsymbol{H}_0(\textrm{div},\Omega ) \cap \boldsymbol{L}^3(\Omega )$, and $\boldsymbol{\chi }\in \boldsymbol{H}(\boldsymbol{\textrm{curl}},\Omega ) \cap \boldsymbol{L}^3(\Omega )$ , with $\textrm{div}\,\boldsymbol{\chi }= 0$.

We are interested in deriving a variational formulation of (3). We introduce the spaces

5 $$\begin{aligned} \boldsymbol{V}:= \boldsymbol{H}^1_0(\Omega )\,, \quad \boldsymbol{W}:= \boldsymbol{H}(\boldsymbol{\textrm{curl}}, \Omega )\,, \quad Q:= L^2_0(\Omega ) \,, \end{aligned}$$

that represent the velocity fields space, the magnetic induction space, and the space of pressures, respectively. Contrary to the convex setting, we emphasize that an $\boldsymbol{H}^1$-conforming space for the magnetic induction cannot be assumed. In the convex case, controlling both the divergence and the $\boldsymbol{\textrm{curl}}$ of a function ensures control over its gradient, which implies that the function belongs to $\boldsymbol{H}^1(\Omega )$. However, when the domain is non-convex, this control is weaker, and we can only conclude that the function lies in $\boldsymbol{H}^{s}(\Omega )$ for some suitable $s > \frac{1}{2}$ depending on $\Omega$. In the velocity space, we introduce the following bilinear forms

6 $$\begin{aligned} a^\textrm{S}(\boldsymbol{u}, \boldsymbol{v}) := (\boldsymbol{\varepsilon }(\boldsymbol{u}), \, \boldsymbol{\varepsilon }(\boldsymbol{v}) ) \,, \qquad c(\boldsymbol{u}, \boldsymbol{v}) := \left( ( \boldsymbol{\nabla }\boldsymbol{u}) \, \boldsymbol{\chi },\, \boldsymbol{v}\right) \,, \end{aligned}$$

where the first one is obtained by multiplying the term $\textrm{div}(\boldsymbol{\varepsilon }(\boldsymbol{u}))$ by a test function $\boldsymbol{v}$, integrating by parts and using the boundary conditions. On $\boldsymbol{W}$, we define the form

$$ a^\textrm{M}(\boldsymbol{B}, \boldsymbol{H}) = (\boldsymbol{\textrm{curl}}(\boldsymbol{B}), \boldsymbol{\textrm{curl}}(\boldsymbol{H})) \, . $$

The coupling is represented by two forms: the first one controls the interaction between velocity and pressure, while the second one governs the coupling between velocity and magnetic induction

7 $$\begin{aligned} \begin{aligned} b(\boldsymbol{v}, q) := (\textrm{div}\boldsymbol{v}, \, q) \,, \qquad d(\boldsymbol{H}, \boldsymbol{v}) := ( \boldsymbol{\textrm{curl}}(\boldsymbol{H}) \times \boldsymbol{\Theta },\, \boldsymbol{v}) \,. \end{aligned} \end{aligned}$$

We also introduce the kernel of the bilinear form $b(\cdot ,\cdot )$ that corresponds to the functions in $\boldsymbol{V}$ with vanishing divergence

8 $$\begin{aligned} \boldsymbol{Z}:= \{ \boldsymbol{v}\in \boldsymbol{V}\quad \text {s.t.} \quad \textrm{div}\, \boldsymbol{v}= 0 \}\,. \end{aligned}$$

Then, we consider the following variational problem

9 $$\begin{aligned} \left\{ \begin{aligned}&\text {find } (\boldsymbol{u}, p, \boldsymbol{B}) \in \boldsymbol{V}\times Q\times \boldsymbol{W}, \text { such that} \\&\begin{aligned} \sigma _\textrm{S}\, (\boldsymbol{u}, \boldsymbol{v}) + \nu _\textrm{S}\, a^\textrm{S}(\boldsymbol{u}, \boldsymbol{v}) + c(\boldsymbol{u}, \boldsymbol{v}) -d(\boldsymbol{B}, \boldsymbol{v}) + b(\boldsymbol{v}, p)&= (\boldsymbol{f}, \boldsymbol{v})&\,\,\,&\text {for all } \boldsymbol{v}\in \boldsymbol{V}, \\ b(\boldsymbol{u}, q)&= 0&\,\,\,&\text {for all } q \in Q, \\ \sigma _\textrm{M}\, (\boldsymbol{B}, \boldsymbol{H}) + \nu _\textrm{M}\, a^\textrm{M}(\boldsymbol{B}, \boldsymbol{H}) + d(\boldsymbol{H}, \boldsymbol{u})&= (\boldsymbol{G}, \boldsymbol{H})&\,\,\,&\text {for all } \boldsymbol{H}\in \boldsymbol{W}. \end{aligned} \end{aligned} \right. \end{aligned}$$

As usual, we suppose that $\boldsymbol{G}$ is $\boldsymbol{L}^2$-orthogonal to the gradients. We also remark that the boundary conditions on $\boldsymbol{B}$, see (4), are here weakly imposed. This problem is well posed by the Lax-Milgram lemma and standard theory of mixed problems.

## Notations and Preliminary Results

In this section, we introduce the notation and some useful results that will be used throughout the rest of the paper. First of all, in order to avoid issues related to the approximation of the domain, we assume that $\Omega$ is a polyhedron. We then consider a family of decompositions $\{ \Omega _h\}_h$ of the domain $\Omega$ into non-overlapping tetrahedrons *E*. We denote with $h_E$ the diameter of each element and with $h:= \max _{E \in \Omega _h} h_E$ the mesh size of $\Omega _h$. Let $\mathcal {N}_h$ be the set of vertices in $\Omega _h$; given $\zeta \in {\mathcal {N}}_h$, we denote with $\omega _{\boldsymbol{\zeta }}$ the collection of elements *E* in $\Omega _h$ such that $\zeta \in \partial E$. We adopt the same mesh assumptions as in [6]. The first assumption is classical in the FEM framework. It states that the elements in the decomposition are not excessively stretched, which is necessary to achieve optimal approximation results.

**(MA1) Shape regularity assumption:**

The mesh family $\left\{ \Omega _h \right\} _h$ is shape regular: it exists a positive constant $c_\mathrm{{M}}$ such that each element $E \in \{ \Omega _h \}_h$ is star shaped with respect to a ball of radius $\varrho _E$ with $h_E \le c_\mathrm{{M}} \varrho _E$.

The second assumption is specific to the case $k=1$. While this assumption is required for theoretical purposes, it is not overly restrictive.

**(MA2) Mesh agglomeration with stars macroelements:**

There exists a family of conforming meshes $\{ \widetilde{\Omega }_h \}_h$ of $\Omega$ with the following properties: (i) it exists a positive constant $\widetilde{c}_\mathrm{{M}}$ such that each element $M \in \widetilde{\Omega }_h$ is a finite (connected) agglomeration of elements in $\Omega _h$, i.e., it exists $\Omega _h^{M}\subset \Omega _h$ with $\mathrm{{card}}(\Omega _h^{M}) \le \widetilde{c}_\mathrm{{M}}$ and $M = \cup _{E \in \Omega _h^{M}} E$; (ii) for any $M \in \widetilde{\Omega }_h$ it exists $\boldsymbol{\zeta }\in \mathcal {N}_h$ such that $\omega _{\boldsymbol{\zeta }}\subseteq M$.

We observe that assumption **(MA1)** readily implies that the mesh is locally quasi-uniform. This means that the diameter of a face is uniformly bounded by the diameters of the elements containing the face and that the sizes of two adjacent elements are comparable.

The set of all the faces in the mesh $\Omega _h$ is denoted by $\Sigma _h$. This set is divided into internal faces $\Sigma _h^\mathrm{{int}}$ and boundary faces $\Sigma _h^{\partial }$. Given an element $E\in \Omega _h$, the set of faces of that element is denoted with $\Sigma _h^E$. Furthermore, given an element $E \in \Omega _h$, we denote with $\boldsymbol{n}^E$ the unit outward normal to that element, while $\boldsymbol{n}^f$ denotes the unit normal to a face *f*. In particular, if $f \in \Sigma _h^\mathrm{{int}}$ we have that $\boldsymbol{n}^f = \boldsymbol{n}^E$ or $\boldsymbol{n}^f = - \boldsymbol{n}^E$, while if $f \in \Sigma _h^{\partial }$ it holds $\boldsymbol{n}^f = \boldsymbol{n}^E = \boldsymbol{n}.$ The jump and the average operators on $f \in \Sigma _h^\mathrm{{int}}$ are defined for every piecewise continuous function w.r.t. $\Omega _h$ respectively by

$$ \begin{aligned} {}[\![\,\varphi \,]\!]_f(\boldsymbol{x})&:= \lim _{s \rightarrow 0^+} \left( \varphi (\boldsymbol{x}- s \boldsymbol{n}_f) - \varphi (\boldsymbol{x}+ s \boldsymbol{n}_f) \right) \\ \left\{ \!\left\{ \,\varphi \,\right\} \!\right\} _f(\boldsymbol{x})&:= \frac{1}{2}\lim _{s \rightarrow 0^+} \left( \varphi (\boldsymbol{x}- s \boldsymbol{n}_f) + \varphi (\boldsymbol{x}+ s \boldsymbol{n}_f) \right) \end{aligned} $$

and $[\![\,\varphi \,]\!]_f(\boldsymbol{x}) = \left\{ \!\left\{ \,\varphi \,\right\} \!\right\} _f(\boldsymbol{x}) = \varphi (\boldsymbol{x})$ on $f \in \Sigma _h^{\partial }$. Given a positive integer $m \in \mathbb {N}$ and a subset $S \subseteq \Omega _h$, we introduce the standard polynomial spaces:

- $\mathbb {P}_m(\omega )$ is the set of polynomials on $\omega$ of degree $\le m$, with $\omega$ a generic set;
- $\mathbb {P}_m(S):= \{q \in L^2\bigl (\cup _{E \in S}E \bigr ) \quad \text {s.t.} \quad q|_{E} \in \mathbb {P}_m(E) \quad \text {for all } E \in S\}$ is the set of piecewise discontinuous polynomials;
- $\mathbb {P}^\textrm{cont}_m(S):= \mathbb {P}_m(S) \cap C^0\bigl (\cup _{E \in S}E \bigr )$ is the set of piecewise continuous polynomial.

Given $s \in \mathbb {R}^+$ and $p \in [1,+\infty ]$, we define the standard broken Sobolev spaces as:

- $$W^s_p(\Omega _h) := \{\varphi \in L^2(\Omega ) \quad \text {s.t.} \quad \varphi |_{E} \in W^s_p(E) \quad \text {for all } E \in \Omega _h\}$$,

equipped with the usual broken norm $\Vert \cdot \Vert _{W^s_p(\Omega _h)}$ and seminorm $\vert \cdot \vert _{W^s_p(\Omega _h)}$. Similarly, we denote with $\boldsymbol{H}^{r}(\boldsymbol{\textrm{curl}},\Omega _h)$ the space of vectorial ${\boldsymbol{L}}^2(\Omega )$ functions $\boldsymbol{H}$ such that $\boldsymbol{H}_{|E}\in \boldsymbol{H}^{r}(\boldsymbol{\textrm{curl}},E)$ for every $E\in \Omega _h$, endowed with the corresponding broken norm. Furthermore, we introduce the following space

- $\mathbb {O}_{k-1}(\Omega _h) := \mathbb {P}^\textrm{cont}_{k-1}(\Omega _h)$ for $k>1$, or $\mathbb {O}_{k-1}(\Omega _h):= \mathbb {P}_{0}(\widetilde{\Omega }_h)$ for $k=1$.

Fix a face $f\in \Sigma _h^\mathrm{{int}}$ shared by two elements $E^+$ and $E^-$. For any $p \in [1,\infty ]$, we will use the notation

$$ \Vert V\Vert _{L^{p}(f^\pm )} := \max \{ \Vert V\Vert _{L^{p}(f^+)}, \Vert V\Vert _{L^{p}(f^-)} \} $$

to denote the $L^p$ norm of a function that is not continuous across *f*. Above $f^+$ (resp., $f^-$) is the face *f* considered as part of the boundary $\partial E^+$ (resp., $\partial E^-$). In the paper, the symbols $\gtrsim$ and $\lesssim$ denote inequalities that hold up to a constant depending solely on the order of the method *k*, the domain $\Omega$, and the regularity of the mesh $\Omega _h$. This constant does not depend on the model parameters $\sigma _\textrm{M}$, $\sigma _\textrm{S}$, $\nu _\textrm{S}$, $\nu _\textrm{M}$, $\boldsymbol{\chi }$, and $\boldsymbol{\Theta }$, nor on the loadings $\textbf{f}$, $\boldsymbol{G}$, or the solution $(\boldsymbol{u}, p, \boldsymbol{B})$.

We conclude this section by mentioning some useful preliminary results, which are well known and can be found for instance in [12, 18] .

### Lemma 1

(Trace inequality) Under the mesh assumption **(MA1)**, for any $E \in \Omega _h$ and for any function $v \in H^s(E)$ with $\frac{1}{2} < s \le 1$, it holds

$$ \sum _{f \in \Sigma _h^E}\Vert v\Vert ^2_{f} \lesssim h_E^{-1}\Vert v\Vert ^2_{E} + h_E^{2s-1}| v|^2_{s,E} \,. $$

### Lemma 2

(Bramble-Hilbert) Under the mesh assumption **(MA1)**, let $m \in {{\mathbb {N}}}$. We denote with $\Pi _{m} :L^2(\Omega _h) \rightarrow \mathbb {P}_m(\Omega _h)$ the $L^2$-projection operator onto the space of piecewise polynomial functions. For any $E \in \Omega _h$ and for any smooth enough function $\varphi$ defined on $\Omega$, it holds

$$ \Vert \varphi - \Pi _{m} \varphi \Vert _{W^r_p(E)} \lesssim h_E^{s-r} \vert \varphi \vert _{W^s_p(E)} \qquad s,r \in \mathbb {N}, r \le s \le m+1, p \in [1, \infty ]. $$

### Lemma 3

(Inverse estimate) Under the mesh assumption **(MA1)**, let $m \in {{\mathbb {N}}}$. Then for any $E \in \Omega _h$ and for any $p_m \in \mathbb {P}_m(E)$ it holds

$$ \Vert p_m\Vert _{W^s_p(E)} \lesssim h_E^{-s} \Vert p_m\Vert _{L^p(E)} $$

where the involved constant only depends on $m,s,p,c_\mathrm{{M}}$.

We consider an interpolation operator that maps sufficiently smooth piecewise discontinuous functions into the space of more regular functions. This interpolation operator is commonly referred to in the literature as an averaging operator [6, 13, 17]. Specifically, our goal is to take a piecewise polynomial *p* of degree $k-1$ and return a piecewise polynomial of the same degree that belongs to $\mathbb {O}_{k-1}(\Omega _h)$. The proof of the following proposition can be found in [6].

### Proposition 4

Let Assumption **(MA1)** hold. Furthermore, if $k=1$ let also Assumption **(MA2)** hold. Then, it exists a projection operator $\mathcal {I}_{\mathcal {O}}:\mathbb {P}_{k-1}(\Omega _h) \rightarrow \mathbb {O}_{k-1}(\Omega _h)$ such that for any $p_{k-1} \in \mathbb {P}_{k-1}(\Omega _h)$ the following holds:

$$ \sum _{E \in \Omega _h}h_E^2 \Vert (I- \mathcal {I}_{\mathcal {O}}) p_{k-1} \Vert _E^2 \lesssim \sum _{f \in \Sigma _h^\mathrm{{int}}} h_f^3 \Vert [\![\,p_{k-1}\,]\!]_f\Vert _f^2 \, . $$

Furthermore, using a triangular inequality combined with a trace inequality, and the regularity of the mesh, it follows

$$ \sum _{E \in \Omega _h}h_E^2 \Vert \mathcal {I}_{\mathcal {O}}p_{k-1} \Vert _E^2 \lesssim \sum _{E \in \Omega _h} h_E^2 \Vert p_{k-1}\Vert _E^2 \, . $$

## The Stabilized Finite Element Method

### Discrete Spaces

Given a positive integer *k* that represents the order of the method, we introduce the following polynomial spaces

10 $$\begin{aligned} \begin{aligned} \boldsymbol{V}^h_k:= [\mathbb {P}_k(\Omega _h)]^3 \cap&\boldsymbol{H}_0(\textrm{div},\Omega ) \, , \quad \boldsymbol{W}^h_k:= [\mathbb {P}_k(\Omega _h)]^3 \cap \boldsymbol{W}\, , \\&Q^h_{k}:= \mathbb {P}_{k-1}(\Omega _h) \cap Q \, . \end{aligned} \end{aligned}$$

For the discrete velocity field $\boldsymbol{V}^h_k$, we select standard $\boldsymbol{H}(\textrm{div})$-conforming Brezzi-Douglas-Marini $\boldsymbol{\textrm{BDM}}_k$ elements, while for the magnetic induction we select Nédéléc elements of the second kind. We introduce the discrete kernel space

$$ \boldsymbol{Z}^h_k:= \{ \boldsymbol{v}_h \in \boldsymbol{V}^h_k\quad \text {s.t.} \quad \textrm{div}\, \boldsymbol{v}_h = 0 \}\, , $$

and thanks to the choice of the $\boldsymbol{\textrm{BDM}}_k$ elements that maintain the pressure robustness of the method, we have the inclusion $\boldsymbol{Z}^h_k\subseteq \boldsymbol{Z}$. We have to introduce two interpolation operators. The first one maps functions in $\boldsymbol{H}^1(\Omega )$ into the space $\boldsymbol{V}^h_k$, mapping the continuous kernel into the discrete kernel, see for instance [9, 18].

#### Lemma 5

(Interpolation operator on $\boldsymbol{V}^h_k$) Under the Assumption **(MA1)** let $\mathcal {I}_{\boldsymbol{V}}:\boldsymbol{V}\rightarrow \boldsymbol{V}^h_k$ be the standard degree-of-freedom interpolation operator defined in the BDM space. Then:

(*i*) if $$\boldsymbol{v}\in \boldsymbol{Z}$$ then $$\mathcal {I}_{\boldsymbol{V}}\boldsymbol{v}\in \boldsymbol{Z}^h_k$$;

(*ii*) for any $\boldsymbol{v}\in \boldsymbol{Z}$

11 $$\begin{aligned} \left( \boldsymbol{v}- \, \mathcal {I}_{\boldsymbol{V}}\boldsymbol{v}, \, \boldsymbol{p}_{k-1} \right) = 0 \quad \text {for all } \boldsymbol{p}_{k-1} \in [\mathbb {P}_{k-1}(\Omega _h)]^3; \end{aligned}$$

(*iii*) for any $\boldsymbol{v}\in \boldsymbol{V}\cap \boldsymbol{H}^{s+1}(\Omega _h)$, with $0 \le s \le k$, for all $E \in \Omega _h$, it holds

12 $$\begin{aligned} \vert \boldsymbol{v}- \mathcal {I}_{\boldsymbol{V}}\boldsymbol{v}\vert _{m,E} \lesssim h_E^{s+1-m} \vert \boldsymbol{v}\vert _{s+1,E} \qquad \text {for } 0\le m\le s+1. \end{aligned}$$

The second interpolation concerns the spaces $\boldsymbol{W}$, see for instance [1]. A notable property is that this interpolant also approximates the $\boldsymbol{\textrm{curl}}$ of the function.

#### Lemma 6

(Interpolation operator on $\boldsymbol{W}^h_k$) Under the Assumption **(MA1)** let $\mathcal {I}_{\boldsymbol{W}}:\boldsymbol{W}\rightarrow \boldsymbol{W}^h_k$ be the interpolation operator of [1]. If the function satisfies also $\boldsymbol{H}\in \boldsymbol{H}^r(\boldsymbol{\textrm{curl}}, \Omega )$ for $\frac{1}{2} < r \le k+1$, the following estimates hold

$$ \begin{aligned}&| \boldsymbol{H}- \mathcal {I}_{\boldsymbol{W}}\boldsymbol{H}|_{m,E} \lesssim h_E^{r-m} | \boldsymbol{H}|_{\boldsymbol{H}^r(\boldsymbol{\textrm{curl}}, E)} \quad&\text {for } 0\le m\le r\, , \\&| \boldsymbol{\textrm{curl}}(\boldsymbol{H}- \mathcal {I}_{\boldsymbol{W}}\boldsymbol{H}) |_{m,E} \lesssim h_E^{\tilde{r}-m}| \boldsymbol{\textrm{curl}}(\boldsymbol{H}) |_{\tilde{r}, E} \quad&\text {for } 0\le m\le {\tilde{r}}\, , \end{aligned} $$

where $${\tilde{r}}:= \min \{ r,k \}$$.

### Discrete Problem

From now on, we assume that the advective fields satisfy the following regularity requirements, in addition to the minimal conditions detailed at the beginning of Section 2:

- the advective velocity field $\boldsymbol{\chi }\in \boldsymbol{C}^0(\overline{\Omega })$;
- the advective magnetic induction $\boldsymbol{\Theta }\in \boldsymbol{C}^1(\overline{\Omega })$.

In addition, before introducing the discrete problem, we preliminary make the following assumption on the solution $\boldsymbol{u}$ of problem (9).

**(RA1) Regularity assumption for the consistency:**

Let $\boldsymbol{u}\in \boldsymbol{Z}$ be the velocity solution of Problem (9), then $\boldsymbol{u}$ belongs to $\boldsymbol{H}^{r}(\Omega )$ for some $r> 3/2$.

We consider the following discrete problem

13 $$\begin{aligned} \left\{ \begin{aligned}&\text {find } (\boldsymbol{u}_h, p_h, \boldsymbol{B}_h) \in \boldsymbol{V}^h_k\times Q^h_{k}\times \boldsymbol{W}^h_k, \text { such that} \\&\begin{aligned} \sigma _\textrm{S}\,(\boldsymbol{u}_h, \boldsymbol{v}_h) + \nu _\textrm{S}\, a^\textrm{S}_h(\boldsymbol{u}_h, \boldsymbol{v}_h) + c_h(\boldsymbol{u}_h, \boldsymbol{v}_h) +&\\ -d(\boldsymbol{B}_h, \boldsymbol{v}_h) + J_h(\boldsymbol{u}_h, \boldsymbol{v}_h) + b(\boldsymbol{v}_h, p_h)&= (\boldsymbol{f}, \boldsymbol{v}_h)&\,\,\,&\text {for all } \boldsymbol{v}_h \in \boldsymbol{V}^h_k, \\ b(\boldsymbol{u}_h, q_h)&= 0&\,\,\,&\text {for all } q_h \in Q^h_{k}, \\ \sigma _\textrm{M}\,(\boldsymbol{B}_h, \boldsymbol{H}_h) + \nu _\textrm{M}\,a^\textrm{M}(\boldsymbol{B}_h, \boldsymbol{H}_h) + d(\boldsymbol{H}_h, \boldsymbol{u}_h)&= (\boldsymbol{G}, \boldsymbol{H}_h)&\,\,\,&\text {for all } \boldsymbol{H}_h \in \boldsymbol{W}^h_k, \end{aligned} \end{aligned} \right. \end{aligned}$$

where the discrete bilinear forms are defined as:

14 $$\begin{aligned} \begin{aligned} a^\textrm{S}_h(\boldsymbol{u}_h, \boldsymbol{v}_h)&:= (\boldsymbol{\varepsilon }_h(\boldsymbol{u}_h) ,\, \boldsymbol{\varepsilon }_h(\boldsymbol{v}_h)) - \sum _{f \in \Sigma _h} (\left\{ \!\left\{ \,\boldsymbol{\varepsilon }_h(\boldsymbol{u}_h)\boldsymbol{n}_f\,\right\} \!\right\} _f ,\, [\![\,\boldsymbol{v}_h\,]\!]_f)_f + \\&\qquad - \sum _{f \in \Sigma _h} ([\![\,\boldsymbol{u}_h\,]\!]_f ,\, \left\{ \!\left\{ \,\boldsymbol{\varepsilon }_h(\boldsymbol{v}_h) \boldsymbol{n}_f\,\right\} \!\right\} _f)_f + \mu _a\sum _{f \in \Sigma _h} h_f^{-1} ([\![\,\boldsymbol{u}_h\,]\!]_f ,\,[\![\,\boldsymbol{v}_h\,]\!]_f)_f \, , \\ c_h(\boldsymbol{u}_h, \boldsymbol{v}_h)&:= (( \boldsymbol{\nabla }_h \boldsymbol{u}_h ) \, \boldsymbol{\chi }, \, \boldsymbol{v}_h ) - \sum _{f \in \Sigma _h^\mathrm{{int}}} ( (\boldsymbol{\chi }\cdot \boldsymbol{n}_f) [\![\,\boldsymbol{u}_h\,]\!]_f ,\, \left\{ \!\left\{ \,\boldsymbol{v}_h\,\right\} \!\right\} _f)_f + \\&\qquad + \mu _c\sum _{f \in \Sigma _h^\mathrm{{int}}} (\vert \boldsymbol{\chi }\cdot \boldsymbol{n}_f \vert [\![\,\boldsymbol{u}_h\,]\!]_f, \, [\![\,\boldsymbol{v}_h\,]\!]_f )_f \, . \end{aligned} \end{aligned}$$

To stabilize the method and obtain feasible numerical solutions in the hyperbolic limit, we include the following jump penalization term in the formulation of the discrete problem

15 $$\begin{aligned} \begin{aligned} J_h(\boldsymbol{u}_h, \boldsymbol{v}_h)&:= \sum _{f \in \Sigma _h} \mu _{J_1}\bigl ( [\![\,\boldsymbol{\Theta }\times \boldsymbol{u}_h\,]\!]_f , [\![\,\boldsymbol{\Theta }\times \boldsymbol{v}_h\,]\!]_f \bigr )_f \\&\qquad + \sum _{f \in \Sigma _h^\mathrm{{int}}} \mu _{J_2}h_f^2 \bigl ([\![\,\boldsymbol{\textrm{curl}}_h (\boldsymbol{u}_h \times \boldsymbol{\Theta })\,]\!]_f ,[\![\,\boldsymbol{\textrm{curl}}_h (\boldsymbol{v}_h \times \boldsymbol{\Theta })\,]\!]_f \bigr )_f \, , \end{aligned} \end{aligned}$$

where $\mu _{J_1}$ and $\mu _{J_2}$ are two parameters that will be fixed later. In contrast to the standard CIP term commonly found in the literature [6, 13], we emphasize that our approach considers the jump of the $\boldsymbol{\textrm{curl}}$ rather than the jump of the full gradient.

#### Remark 7

It is possible to consider the following alternative $J_h(\cdot ,\cdot )$:

$$ \begin{aligned} J_h(\boldsymbol{u}_h, \boldsymbol{v}_h)&:= \sum _{f \in \Sigma _h} \Vert \boldsymbol{\Theta }\Vert _{\boldsymbol{L}^\infty ({f})}^2 \mu _{J_1}([\![\,\boldsymbol{u}_h\,]\!]_f , [\![\,\boldsymbol{v}_h\,]\!]_f)_f \\&\qquad + \sum _{f \in \Sigma _h^\mathrm{{int}}} \Vert \boldsymbol{\Theta }\Vert _{\boldsymbol{W}^1_\infty ({f})}^2 \mu _{J_2}h_f^2 ([\![\,\boldsymbol{\nabla }_h \boldsymbol{u}_h\,]\!]_f , [\![\,\boldsymbol{\nabla }_h \boldsymbol{v}_h\,]\!]_f )_f \, . \end{aligned} $$

Finally, we define the bilinear form

16 $$\begin{aligned} \begin{aligned} \mathcal {A}_\textrm{stab}(\boldsymbol{u}_h, \boldsymbol{B}, \boldsymbol{v}_h, \boldsymbol{H}_h) :=&\sigma _\textrm{S}(\boldsymbol{u}_h, \boldsymbol{v}_h) + \sigma _\textrm{M}(\boldsymbol{B}, \boldsymbol{H}_h) + \nu _\textrm{S}a^\textrm{S}_h(\boldsymbol{u}_h, \boldsymbol{v}_h) + \nu _\textrm{M}a^\textrm{M}(\boldsymbol{B}, \boldsymbol{H}_h) \\&+ c_h(\boldsymbol{u}_h, \boldsymbol{v}_h) -d(\boldsymbol{B}, \boldsymbol{v}_h) + d(\boldsymbol{H}_h, \boldsymbol{u}_h) + J_h(\boldsymbol{u}_h, \boldsymbol{v}_h) \,. \end{aligned} \end{aligned}$$

Problem (13) can be written also in the following pressure-independent form

17 $$\begin{aligned} \left\{ \begin{aligned}&\text {find } (\boldsymbol{u}_h,\boldsymbol{B}_h) \in \boldsymbol{Z}^h_k\times \boldsymbol{W}^h_k, \text { such that} \\&\mathcal {A}_\textrm{stab}(\boldsymbol{u}_h, \boldsymbol{B}_h, \boldsymbol{v}_h, \boldsymbol{H}_h) = (\boldsymbol{f}, \boldsymbol{v}_h) + (\boldsymbol{G}, \boldsymbol{H}_h) \quad \text {for all } \boldsymbol{v}_h \in \boldsymbol{Z}^h_k, \, \text {for all } \boldsymbol{H}_h \in \boldsymbol{W}^h_k\, . \end{aligned} \right. \end{aligned}$$

#### Remark 8

All the forms above are intended to be extendable to any sufficiently regular function. In particular, if the solution $\boldsymbol{u}$ of the continuous problem (9) satisfies (RA1), the following consistency property holds

18 $$\begin{aligned} \mathcal {A}_\textrm{stab}(\boldsymbol{u}-\boldsymbol{u}_h, \boldsymbol{B}-\boldsymbol{B}_h, \boldsymbol{v}_h, \boldsymbol{H}_h) = 0 \quad \text {for all } \boldsymbol{v}_h \in \boldsymbol{Z}^h_k, \, \text {for all } \boldsymbol{H}_h \in \boldsymbol{W}^h_k\, . \end{aligned}$$

#### Remark 9

Exploiting the third equation in (13), thanks to the assumption that $\boldsymbol{G}$ is orthogonal to the gradients, and choosing $\boldsymbol{H}_h$ as the gradient of a polynomial $p \in \mathbb {P}^\textrm{cont}_{k+1}(\Omega _h)$, we obtain that

$$ (\boldsymbol{B}_h, \nabla p) = 0 \quad \forall p \in \mathbb {P}^\textrm{cont}_{k+1}(\Omega _h) \, , $$

which is the classical discrete divergence-free condition for Nédélec elements.

## Theoretical Analysis

### Inf-sup Condition

We introduce the following norms and seminorms, which depend on the equation parameters and on the mesh:

19 $$\begin{aligned} \begin{aligned} \left\| \boldsymbol{u}\right\| _\textrm{S}^2&:= \sigma _\textrm{S}\Vert \boldsymbol{u}\Vert ^2 + \nu _\textrm{S}\Vert \boldsymbol{\varepsilon }_h(\boldsymbol{u}) \Vert ^2 + \nu _\textrm{S}\, \mu _a\!\sum _{f \in \Sigma _h} h_f^{-1} \Vert [\![\,\boldsymbol{u}\,]\!]_f \Vert _{f}^2 \, , \\ \left| \boldsymbol{u}\right| _\textrm{upw}^2&:= \mu _c\!\sum _{f \in \Sigma _h^\mathrm{{int}}} \Vert \vert \boldsymbol{\chi }\cdot \boldsymbol{n}_f \vert ^{1/2} [\![\,\boldsymbol{u}\,]\!]_f \Vert _{f}^2 \, , \\ \left| \boldsymbol{u}\right| _\textrm{cip}^2&:= \mu _{J_1}\sum _{f \in \Sigma _h} \Vert [\![\,\boldsymbol{\Theta }\times \boldsymbol{u}\,]\!]_f \Vert _{f}^2 \, , + \mu _{J_2}\sum _{f \in \Sigma _h^\mathrm{{int}}} h_f^2 \Vert [\![\,\boldsymbol{\textrm{curl}}_h(\boldsymbol{u}\times \boldsymbol{\Theta }_h)\,]\!]_f \Vert _{f}^2\\ \left| \boldsymbol{u}\right| _\mathrm{\boldsymbol{\textrm{curl}}}^2&:= \gamma ^{-1} \, \sum _{E \in \Omega _h} h_E^2 \, \Vert \boldsymbol{\textrm{curl}}_h(\boldsymbol{u}\times \boldsymbol{\Theta }_h) \Vert ^2_{E} \, . \end{aligned} \end{aligned}$$

where the global parameter $\gamma := \max \{h, \nu _\textrm{M}\}$ (hence $\gamma ^{-1}:= \min \{h^{-1},\nu _\textrm{M}^{-1}\}$) and $\boldsymbol{\Theta }_h$ is the best piecewise constant approximation of $\boldsymbol{\Theta }$ in $L^2(\Omega )$.

#### Remark 10

We are aware that *h* and $\nu _\textrm{M}$ have different physical dimensions, so that in principle the comparison $\gamma := \max \{h, \nu _\textrm{M}\}$ would require a suitable preliminary scaling of these quantities. However, it is common practice in the literature concerning finite element analysis of advection-diffusion-reaction problems to ignore this aspect and assume that the involved quantities are already properly scaled. Here, we follow this approach.

The stability norms are obtained by summing these norms and seminorms

20 $$\begin{aligned} \begin{aligned} \left\| \boldsymbol{u}\right\| _\textrm{stab}^2&:= \left\| \boldsymbol{u}\right\| _\textrm{S}^2 + \left| \boldsymbol{u}\right| _\textrm{upw}^2 + \left| \boldsymbol{u}\right| _\textrm{cip}^2 + \left| \boldsymbol{u}\right| _\mathrm{\boldsymbol{\textrm{curl}}}^2 \,, \\ \left\| \boldsymbol{B}\right\| _\textrm{M}^2&:= \sigma _\textrm{M}\Vert \boldsymbol{B}\Vert ^2 + \nu _\textrm{M}\Vert \boldsymbol{\textrm{curl}}(\boldsymbol{B}) \Vert ^2 \,. \end{aligned} \end{aligned}$$

#### Remark 11

In the definitions of the seminorm $\left| \cdot \right| _\mathrm{\boldsymbol{\textrm{curl}}}$ and the second term in $\left| \cdot \right| _\textrm{cip}$, we have considered the approximation $\boldsymbol{\Theta }_h$ instead of $\boldsymbol{\Theta }$. This choice was made to simplify the theoretical analysis of the method. We emphasize that controlling the norms with $\boldsymbol{\Theta }_h$, along with the ${\boldsymbol{L}}^2$-norm of $\boldsymbol{v}_h$, ensures the control of the corresponding norms with $\boldsymbol{\Theta }$ as we show briefly here below (and also the converse holds true). In fact, using triangular inequality, an inverse estimate and standard approximation results together with inverse estimates for polynomials, we obtain

$$ \begin{aligned} \Vert \boldsymbol{\textrm{curl}}_h(\boldsymbol{v}_h \times \boldsymbol{\Theta }_h) \Vert ^2_{E}&\le \Vert \boldsymbol{\textrm{curl}}_h(\boldsymbol{v}_h \times \boldsymbol{\Theta }) \Vert ^2_{E} + \Vert \boldsymbol{\textrm{curl}}_h(\boldsymbol{v}_h \times (\boldsymbol{\Theta }_h - \boldsymbol{\Theta })) \Vert ^2_{E} \\&\lesssim \Vert \boldsymbol{\textrm{curl}}_h(\boldsymbol{v}_h \times \boldsymbol{\Theta }) \Vert ^2_{E} + | \boldsymbol{v}_h |_{1,E}^2\Vert \boldsymbol{\Theta }_h - \boldsymbol{\Theta }\Vert ^2_{\boldsymbol{L}^\infty (E)} \\&\qquad + \Vert \boldsymbol{v}_h \Vert _E^2 | \boldsymbol{\Theta }_h - \boldsymbol{\Theta }|^2_{\boldsymbol{W}^1_\infty (E)}\\&\lesssim \Vert \boldsymbol{\textrm{curl}}_h(\boldsymbol{v}_h \times \boldsymbol{\Theta }) \Vert ^2_{E} + |\boldsymbol{\Theta }|^2_{\boldsymbol{W}^1_\infty (E)}\Vert \boldsymbol{v}_h\Vert ^2_{E} \, , \\ \end{aligned} $$

for all $\boldsymbol{v}_h \in \boldsymbol{Z}^h_k$ (but, obviously, it holds also for all $\boldsymbol{v}_h \in \boldsymbol{V}^h_k$). For the seminorm $\left| \cdot \right| _\textrm{cip}$, we have that

$$ \begin{aligned} \sum _{f\in \Sigma _h^\mathrm{{int}}} h_f^2 \Vert [\![\,\boldsymbol{\textrm{curl}}_h(\boldsymbol{v}_h \times \boldsymbol{\Theta }_h) \,]\!] \Vert ^2_{f}&\le \sum _{f\in \Sigma _h^\mathrm{{int}}} h_f^2 \Vert [\![\,\boldsymbol{\textrm{curl}}_h(\boldsymbol{v}_h \times \boldsymbol{\Theta }) \,]\!] \Vert ^2_{f} \\&\qquad + \sum _{f\in \Sigma _h^\mathrm{{int}}} h_f^2 \Vert [\![\,\boldsymbol{\textrm{curl}}_h(\boldsymbol{v}_h \times (\boldsymbol{\Theta }_h-\boldsymbol{\Theta })) \,]\!] \Vert ^2_{f}\, . \end{aligned} $$

Noting that

$$\begin{aligned} \sum _{f\in \Sigma _h^\mathrm{{int}}} h_f^2 \Vert [\![\,\boldsymbol{\textrm{curl}}_h(\boldsymbol{v}_h \times (\boldsymbol{\Theta }_h-\boldsymbol{\Theta })) \,]\!] \Vert ^2_{f}\le &   \sum _{f\in \Sigma _h^\mathrm{{int}}} h_f^2 \bigl (\Vert \boldsymbol{\textrm{curl}}_h(\boldsymbol{v}_h \times (\boldsymbol{\Theta }_h-\boldsymbol{\Theta })) \Vert ^2_{f^+} \\  &   + \Vert \boldsymbol{\textrm{curl}}_h(\boldsymbol{v}_h \times (\boldsymbol{\Theta }_h-\boldsymbol{\Theta })) \Vert ^2_{f^-} \bigr ) \, , \end{aligned}$$

and using approximation estimates for $\boldsymbol{\Theta }\in \boldsymbol{C}^1(\overline{\Omega })$, a scaled trace inequality and inverse estimates on $\boldsymbol{v}_h$, we easily obtain

21 $$\begin{aligned} \begin{aligned} \sum _{f\in \Sigma _h^\mathrm{{int}}} h_f^2 \Vert [\![\,\boldsymbol{\textrm{curl}}(\boldsymbol{v}_h \times (\boldsymbol{\Theta }_h-\boldsymbol{\Theta })) \,]\!] \Vert ^2_{f}&\lesssim \sum _{f \in \Sigma _h^\mathrm{{int}}}h_f^2 \Vert \nabla \boldsymbol{v}_h \Vert ^2_{\boldsymbol{L}^2(f^\pm )} \Vert \boldsymbol{\Theta }- \boldsymbol{\Theta }_h \Vert ^2_{\boldsymbol{L}^{\infty }(f^\pm )} + \\&\qquad + \sum _{f \in \Sigma _h^\mathrm{{int}}}h_f^2\Vert \boldsymbol{v}_h \Vert ^2_{\boldsymbol{L}^2(f^\pm )} | \boldsymbol{\Theta }|^2_{\boldsymbol{W}^1_{\infty }({f})} \\&\lesssim \sum _{E \in \Omega _h} h_E \Vert \boldsymbol{v}_h \Vert ^2_{E} |\boldsymbol{\Theta }|^2_{\boldsymbol{W}^1_\infty (E)} \lesssim h \,|\boldsymbol{\Theta }|^2_{\boldsymbol{W}^1_\infty ({\Omega })} \Vert \boldsymbol{v}_h \Vert ^2 \, . \end{aligned} \end{aligned}$$

Hence

$$ \sum _{f\in \Sigma _h^\mathrm{{int}}} h_f^2 \Vert [\![\,\boldsymbol{\textrm{curl}}_h(\boldsymbol{v}_h \times \boldsymbol{\Theta }) \,]\!] \Vert ^2_{f} {\le } \sum _{f\in \Sigma _h^\mathrm{{int}}} h_f^2 \Vert [\![\,\boldsymbol{\textrm{curl}}_h(\boldsymbol{v}_h \times \boldsymbol{\Theta }_h) \,]\!] \Vert ^2_{f} {+} \sum _{E \in \Omega _h} h_E | \boldsymbol{\Theta }|^2_{\boldsymbol{W}^1_\infty ({\Omega })} \Vert \boldsymbol{v}_h\Vert ^2_E $$

The proof of the inf-sup stability is divided into two parts. First, we control all the terms appearing in the previous definition, except for $\left| \cdot \right| _\mathrm{\boldsymbol{\textrm{curl}}}$, by testing the bilinear form $\mathcal {A}_\textrm{stab}(\cdot , \cdot , \cdot , \cdot )$ with a symmetric entry. To handle the remaining term, we use a suitable interpolant of a function close to $\boldsymbol{\textrm{curl}}(\cdot \times \boldsymbol{\Theta })$ and construct a function that satisfies a form of inf-sup condition with respect to such interpolant.

We omit the proof of the following result since it can be obtained with a standard argument in DG theory; we only underline that also Remark 11 needs to be used to bound the $\left| \cdot \right| _\textrm{cip}^2$ term in the right-hand side.

#### Proposition 12

Let the mesh assumptions **(MA1)**, and **(MA2)** if $k=1$. Assume the consistency assumption **(RA1)** holds. If the parameter $\mu _a$ in (14) is sufficiently large there exists a real positive constant $c_\textrm{coe}$ such that for all $\boldsymbol{v}_h \in \boldsymbol{Z}^h_k$ and $\boldsymbol{H}_h \in \boldsymbol{W}$ the form $\mathcal {A}_\textrm{stab}(\cdot , \cdot , \cdot , \cdot )$ defined in (16) satisfies

$$ \mathcal {A}_\textrm{stab}(\boldsymbol{v}_h, \boldsymbol{B}_h, \boldsymbol{v}_h, \boldsymbol{B}_h) \ge c_\textrm{coe}\left( \left\| \boldsymbol{v}_h\right\| _\textrm{S}^2 + \left| \boldsymbol{v}_h\right| _\textrm{upw}^2 + \left| \boldsymbol{v}_h\right| _\textrm{cip}^2 + \left\| \boldsymbol{B}_h\right\| _\textrm{M}^2 \right) \, , $$

where the coefficient $c_\textrm{coe}$ does not depend on the mesh size *h* and on the problem parameters $\sigma _\textrm{S}$, $\sigma _\textrm{M}$, $\nu _\textrm{S}$, $\nu _\textrm{M}$, $\boldsymbol{\chi }$, and $\boldsymbol{\Theta }$.

The following result aims to establish control over $\left| \boldsymbol{v}_h\right| _\mathrm{\boldsymbol{\textrm{curl}}}$. We emphasize that, in the previous proposition, we managed to control all the terms in the norm except for $\left| \boldsymbol{v}_h\right| _\mathrm{\boldsymbol{\textrm{curl}}}$.

#### Proposition 13

Let the mesh assumptions **(MA1)**, and **(MA2)** if $k=1$. Assume that the consistency assumption **(RA1)** holds. Then for any $(\boldsymbol{v}_h, \boldsymbol{B}_h) \in \boldsymbol{Z}^h_k\times \boldsymbol{W}^h_k$ it exists $\boldsymbol{H}_h \in \boldsymbol{W}^h_k$ such that

22 $$\begin{aligned} \begin{aligned} \mathcal {A}_\textrm{stab}(\boldsymbol{v}_h, \boldsymbol{B}_h, 0, \boldsymbol{H}_h)&\ge C_1\left| v_h\right| _\mathrm{\boldsymbol{\textrm{curl}}}^2 -C \Big (\mu _{J_2}^{-1} \left| \boldsymbol{v}_h\right| _\textrm{cip}^2 + (1 + \sigma _\textrm{M}h) \left\| \boldsymbol{B}_h\right\| _\textrm{M}^2 \\  &\qquad + \frac{| \boldsymbol{\Theta }|^2_{\boldsymbol{W}^1_\infty ({\Omega })} \, h}{\sigma _\textrm{S}} \Vert \boldsymbol{v}_h \Vert ^2_S + \left( \frac{\mu _{J_1}+ \mu _{J_2}}{\mu _{J_1}\,\mu _{J_2}} \right) \left| \boldsymbol{v}_h\right| _\textrm{cip}^2\Big ) \, , \end{aligned} \end{aligned}$$

where the constants $C_1$ and *C* does not depend on the mesh size *h* and on the problem parameters $\sigma _\textrm{S}$, $\sigma _\textrm{M}$, $\nu _\textrm{S}$, $\nu _\textrm{M}$, $\boldsymbol{\chi }$, and $\boldsymbol{\Theta }$.

#### Proof

We start by constructing $\boldsymbol{H}_h$. We introduce $\boldsymbol{p}_h\in \mathbb {O}_{k-1}(\Omega _h)$ as the interpolant of $\boldsymbol{\textrm{curl}}_h(\boldsymbol{v}_h \times \boldsymbol{\Theta }_h)$ defined in Proposition 4. Let $\hat{\boldsymbol{H}}_h \in \mathbb {P}^\textrm{cont}_k(\Omega _h) \cap \boldsymbol{W}$ be the function constructed in [6] Lemma 4.3 (Equation 4.9) that satisfies the following two inequalities:

23 $$\begin{aligned} \left\{ \begin{aligned}&\sum _{E \in \Omega _h} h_E^{-2} \Vert {\hat{\boldsymbol{H}}}_h \Vert _E^2 \lesssim \sum _{E \in \Omega _h} h_E^{2} \Vert \boldsymbol{p}_h \Vert _E^2 \, , \\&({\hat{\boldsymbol{H}}}_h, \boldsymbol{p}_h) \gtrsim \sum _{E \in \Omega _h} h_E^{2} \Vert \boldsymbol{p}_h \Vert _E^2 \, . \end{aligned} \right. \end{aligned}$$

Finally, we define $\boldsymbol{H}_h = \gamma ^{-1} {\hat{\boldsymbol{H}}}_h$ and proceed to show that it satisfies (22).

We observe that, since the third entry is equal to zero, we easily obtain that

24 $$\begin{aligned} \sigma _\textrm{S}(\boldsymbol{v}_h, 0) = \nu _\textrm{S}\, a^\textrm{S}_h(\boldsymbol{v}_h, 0) = c_h(\boldsymbol{v}_h, 0) = d(\boldsymbol{B}, 0) = J_h(\boldsymbol{v}_h, 0) = 0 \, . \end{aligned}$$

Now, we estimate the nonzero terms. By the Cauchy-Schwarz inequality and recalling that $\boldsymbol{H}_h = \gamma ^{-1} {\hat{\boldsymbol{H}}}_h$, we get

$$\begin{aligned} \begin{aligned} | \sigma _\textrm{M}(\boldsymbol{B}_h, \boldsymbol{H}_h) |&= \big | \sigma _\textrm{M}\sum _{E \in \Omega _h}\int _E \boldsymbol{B}_h\cdot \boldsymbol{H}_h\big | \ge -\sigma _\textrm{M}\left( \sum _{E \in \Omega _h} h_E^2 \, \Vert \boldsymbol{B}_h \Vert ^2_E\right) ^{\frac{1}{2}} \left( \sum _{E \in \Omega _h} h_E^{-2} \, \Vert \boldsymbol{H}_h \Vert ^2_E\right) ^{\frac{1}{2}}\\&= -\sigma _\textrm{M}\left( \sum _{E \in \Omega _h} h_E^2 \, \Vert \boldsymbol{B}_h \Vert ^2_E\right) ^{\frac{1}{2}} \gamma ^{-1}\left( \sum _{E \in \Omega _h} h_E^{-2} \, \Vert \hat{\boldsymbol{H}}_h \Vert ^2_E\right) ^{\frac{1}{2}} \, . \end{aligned} \end{aligned}$$

By the first estimate in (23), recalling that $\boldsymbol{p}_h =\mathcal {I}_{\mathcal {O}}(\boldsymbol{\textrm{curl}}_h(\boldsymbol{v}_h \times \boldsymbol{\Theta }_h))$ (cf. Proposition 4), observing that $h_E\le h$ and using the second estimate in Proposition 4, we have

25 $$\begin{aligned} \begin{aligned} | \sigma _\textrm{M}(\boldsymbol{B}_h, \boldsymbol{H}_h) |&\gtrsim -\sigma _\textrm{M}\left( \sum _{E \in \Omega _h} h_E^2 \, \Vert \boldsymbol{B}_h \Vert ^2_E\right) ^{\frac{1}{2}} \gamma ^{-1} \left( \sum _{E \in \Omega _h} h_E^{2} \, \Vert \boldsymbol{p}_h \Vert ^2_E\right) ^{\frac{1}{2}}\\&= -\sigma _\textrm{M}\left( \sum _{E \in \Omega _h} h_E^2 \, \Vert \boldsymbol{B}_h \Vert ^2_E\right) ^{\frac{1}{2}} \gamma ^{-1} \left( \sum _{E \in \Omega _h} h_E^{2} \, \Vert \mathcal {I}_{\mathcal {O}}(\boldsymbol{\textrm{curl}}_h(\boldsymbol{v}_h \times \boldsymbol{\Theta }_h))\Vert ^2_E\right) ^{\frac{1}{2}}\\&\gtrsim -\sigma _\textrm{M}\, h \, \Vert \boldsymbol{B}_h \Vert \, \gamma ^{-1} \left( \sum _{E \in \Omega _h} h_E^{2} \, \Vert \boldsymbol{\textrm{curl}}_h(\boldsymbol{v}_h \times \boldsymbol{\Theta }_h)\Vert ^2_E\right) ^{\frac{1}{2}} \, . \end{aligned} \end{aligned}$$

By the definitions of the seminorm $\left| \cdot \right| _\mathrm{\boldsymbol{\textrm{curl}}}$ and the norm $\left\| \cdot \right\| _\textrm{M}$, cf. (19) and (20), from (25) we obtain

$$\begin{aligned} | \sigma _\textrm{M}(\boldsymbol{B}_h, \boldsymbol{H}_h) | \gtrsim -\sigma _\textrm{M}^{\frac{1}{2}} \, h \, \left\| \boldsymbol{B}_h\right\| _\textrm{M} \gamma ^{-\frac{1}{2}} \left| \boldsymbol{v}_h\right| _\mathrm{\boldsymbol{\textrm{curl}}} \, . \end{aligned}$$

Therefore, recalling that $\gamma ^{-1}:= \min \{h^{-1},\nu _\textrm{M}^{-1}\}\le h^{-1}$, we get

26 $$\begin{aligned} | \sigma _\textrm{M}(\boldsymbol{B}_h, \boldsymbol{H}_h) | \gtrsim -\sigma _\textrm{M}^{\frac{1}{2}} \, h^{\frac{1}{2}} \, \left\| \boldsymbol{B}_h\right\| _\textrm{M} \left| \boldsymbol{v}_h\right| _\mathrm{\boldsymbol{\textrm{curl}}} \, . \end{aligned}$$

The bilinear form $a^\textrm{M}(\boldsymbol{B}_h,\boldsymbol{H}_h)$ is estimated in a very similar way, by using also a polynomial inverse estimate and the fact that $\gamma \ge \nu _\textrm{M}$. We get

27 $$\begin{aligned} \!\!\!\!\!\! | a^\textrm{M}(\boldsymbol{B}_h, \boldsymbol{H}_h) |\ge &   - \nu _\textrm{M}\Vert \boldsymbol{\textrm{curl}}(\boldsymbol{B}_h) \Vert \Vert \boldsymbol{\textrm{curl}}(\boldsymbol{H}_h) \Vert \nonumber \\\gtrsim &   -\nu _\textrm{M}\left( \sum _{E \in \Omega _h} \Vert \boldsymbol{\textrm{curl}}(\boldsymbol{B}_h) \Vert ^2_E\right) ^{\frac{1}{2}} \left( \sum _{E \in \Omega _h} \Vert \boldsymbol{\textrm{curl}}(\boldsymbol{H}_h) \Vert ^2_E\right) ^{\frac{1}{2}} \nonumber \\\gtrsim &   -\nu _\textrm{M}^{\frac{1}{2}}\left\| \boldsymbol{B}_h\right\| _\textrm{M} \left( \sum _{E \in \Omega _h} h_E^{-2} \, \Vert \boldsymbol{H}_h \Vert ^2_E\right) ^{\frac{1}{2}}\nonumber \\\gtrsim &   -\nu _\textrm{M}^{\frac{1}{2}}\left\| \boldsymbol{B}_h\right\| _\textrm{M} \gamma ^{-1}\!\left( \sum _{E \in \Omega _h} h_E^{-2} \, \Vert \hat{\boldsymbol{H}}_h \Vert ^2_E\right) ^{\frac{1}{2}} \nonumber \\\gtrsim &   -\nu _\textrm{M}^{\frac{1}{2}}\left\| \boldsymbol{B}_h\right\| _\textrm{M} \gamma ^{-1}\left( \sum _{E \in \Omega _h} h_E^2 \, \Vert \boldsymbol{\textrm{curl}}_h(\boldsymbol{v}_h \times \boldsymbol{\Theta }_h) \Vert ^2_E\right) ^{\frac{1}{2}} \gtrsim -\left\| \boldsymbol{B}_h\right\| _\textrm{M} \left| \boldsymbol{v}_h\right| _\mathrm{\boldsymbol{\textrm{curl}}}. \end{aligned}$$

For the bilinear form $d(\boldsymbol{H}_h,\boldsymbol{v}_h)$, thanks to integration by parts, we have that

28 $$\begin{aligned} \begin{aligned} d(\boldsymbol{H}_h, \boldsymbol{v}_h)&= (\boldsymbol{\textrm{curl}}(\boldsymbol{H}_h) \times \boldsymbol{\Theta }, \boldsymbol{v}_h) \\&= (\boldsymbol{\textrm{curl}}_h (\boldsymbol{\Theta }\times \boldsymbol{v}_h), \boldsymbol{H}_h) + \sum _{E \in \Omega _h} (\boldsymbol{H}_h \times \boldsymbol{n}_E , \boldsymbol{\Theta }\times \boldsymbol{v}_h)_{\partial E} \, . \end{aligned} \end{aligned}$$

The first term is split as

$$ \begin{aligned} (\boldsymbol{\textrm{curl}}_h (\boldsymbol{\Theta }\times \boldsymbol{v}_h), \boldsymbol{H}_h)&= (\boldsymbol{\textrm{curl}}_h ((\boldsymbol{\Theta }- \boldsymbol{\Theta }_h) \times \boldsymbol{v}_h) , \boldsymbol{H}_h) + (\boldsymbol{\textrm{curl}}_h (\boldsymbol{\Theta }_h \times \boldsymbol{v}_h) , \boldsymbol{H}_h) \\&= (\boldsymbol{\textrm{curl}}_h ((\boldsymbol{\Theta }- \boldsymbol{\Theta }_h) \times \boldsymbol{v}_h) , \boldsymbol{H}_h) \\&\qquad + (\boldsymbol{\textrm{curl}}_h (\boldsymbol{\Theta }_h \times \boldsymbol{v}_h) - \boldsymbol{p}_h, \boldsymbol{H}_h) \\&\qquad + (\boldsymbol{p}_h, \boldsymbol{H}_h) \\&=: T_{\boldsymbol{\Theta },1} + T_{\boldsymbol{\Theta },2} + T_{\boldsymbol{\Theta },3} \, . \end{aligned} $$

*Estimate of* $T_{\boldsymbol{\Theta },1}$: Thanks to the regularity of $\boldsymbol{\Theta }$, we have that

$$\begin{aligned} \begin{aligned} (\boldsymbol{\textrm{curl}}_h ((\boldsymbol{\Theta }- \boldsymbol{\Theta }_h) \times \boldsymbol{v}_h) , \boldsymbol{H}_h)&\ge - \left( \sum _{E \in \Omega _h} h_E^2 \Vert \boldsymbol{\textrm{curl}}_h ((\boldsymbol{\Theta }- \boldsymbol{\Theta }_h) \times \boldsymbol{v}_h)\Vert _E^2 \right) ^{\frac{1}{2}} \\&\qquad \left( \sum _{E \in \Omega _h} h_E^{-2} \Vert \boldsymbol{H}_h\Vert _E^2 \right) ^{\frac{1}{2}} \, . \end{aligned} \end{aligned}$$

Using the vector calculus identity,

29 $$\begin{aligned} \boldsymbol{\textrm{curl}}(A \times B) = (\textrm{div}B) \, A - (\textrm{div}A) \, B + (\boldsymbol{\nabla }A) B - (\boldsymbol{\nabla }B) A \, , \end{aligned}$$

and a polynomial inverse estimate combined with standard approximation results, we can easily obtain

$$ \Vert \boldsymbol{\textrm{curl}}_h ((\boldsymbol{\Theta }- \boldsymbol{\Theta }_h) \times \boldsymbol{v}_h)\Vert ^2_E \le |\boldsymbol{\Theta }|_{\boldsymbol{W}^1_\infty ({\Omega })} \, \Vert \boldsymbol{v}_h \Vert _E \, . $$

Hence, using the same steps as the previous estimates, we have that

$$ \begin{aligned} (\boldsymbol{\textrm{curl}}_h ((\boldsymbol{\Theta }- \boldsymbol{\Theta }_h) \times \boldsymbol{v}_h) , \boldsymbol{H}_h)&\gtrsim - \left( \sum _{E \in \Omega _h} h_E^2 |\boldsymbol{\Theta }|_{\boldsymbol{W}^1_\infty ({\Omega })} \, \Vert \boldsymbol{v}_h \Vert _E \right) ^{\frac{1}{2}} \\&\qquad \gamma ^{-1}\left( \sum _{E \in \Omega _h} h_E^2 \, \Vert \boldsymbol{\textrm{curl}}_h(\boldsymbol{v}_h \times \boldsymbol{\Theta }_h) \Vert ^2_E\right) ^{\frac{1}{2}} \\&\gtrsim - | \boldsymbol{\Theta }|_{\boldsymbol{W}^1_\infty ({\Omega })} h^{\frac{1}{2}} \Vert \boldsymbol{v}_h \Vert \, \left| \boldsymbol{v}_h\right| _\mathrm{\boldsymbol{\textrm{curl}}} \\&\gtrsim - \left( \frac{| \boldsymbol{\Theta }|^2_{\boldsymbol{W}^1_\infty ({\Omega })} \, h}{\sigma _\textrm{S}}\right) ^{\frac{1}{2}} \Vert \boldsymbol{v}_h \Vert _S \, \left| \boldsymbol{v}_h\right| _\mathrm{\boldsymbol{\textrm{curl}}} \, . \end{aligned} $$

*Estimate of* $T_{\boldsymbol{\Theta },2}$: Using Proposition 4, the definition and properties of $\boldsymbol{H}_h$, and recalling that $h_E \le h$, we obtain

$$ \begin{aligned} (\boldsymbol{\textrm{curl}}_h (\boldsymbol{\Theta }_h \times \boldsymbol{v}_h) - \boldsymbol{p}_h, \boldsymbol{H}_h)&\gtrsim -\left( \sum _{f \in \Sigma _h^\mathrm{{int}}} h_f^3 \Vert [\![\,\boldsymbol{\textrm{curl}}_h(\boldsymbol{\Theta }_h \times \boldsymbol{v}_h)\,]\!]\Vert _f^2\right) ^{\frac{1}{2}} \\&\qquad \gamma ^{-1} \left( \sum _{E \in \Omega _h} h_E^{2} \, \Vert \boldsymbol{\textrm{curl}}_h(\boldsymbol{v}_h \times \boldsymbol{\Theta }_h) \Vert ^2_E\right) ^{\frac{1}{2}} \\&\gtrsim - \left( \frac{1}{\mu _{J_2}}\right) ^{\frac{1}{2}} \left| \boldsymbol{v}_h\right| _\textrm{cip}\left| \boldsymbol{v}_h\right| _\mathrm{\boldsymbol{\textrm{curl}}} \, . \end{aligned} $$

*Estimate of* $T_{\boldsymbol{\Theta },3}$: Using the definition of $\boldsymbol{H}_h$ and the properties of ${\hat{\boldsymbol{H}}}_h$ yields

$$ (\boldsymbol{p}_h, \boldsymbol{H}_h) = \gamma ^{-1} (\boldsymbol{p}_h, {\hat{\boldsymbol{H}}}_h) \gtrsim \gamma ^{-1} \sum _{E \in \Omega _h} h_E^2 \Vert \boldsymbol{p}_h \Vert ^2_E \, . $$

Now, using triangular inequality, we observe that

$$ \Vert \boldsymbol{\textrm{curl}}_h(\boldsymbol{v}_h \times \boldsymbol{\Theta }_h) \Vert ^2_E \le 2\Vert \boldsymbol{p}_h \Vert ^2_E + 2\Vert \boldsymbol{\textrm{curl}}_h(\boldsymbol{v}_h \times \boldsymbol{\Theta }_h) - \boldsymbol{p}_h\Vert ^2_E \, , $$

which implies

$$ \Vert \boldsymbol{p}_h \Vert ^2_E \ge \frac{1}{2}\Vert \boldsymbol{\textrm{curl}}_h(\boldsymbol{v}_h \times \boldsymbol{\Theta }_h) \Vert ^2_E - \Vert \boldsymbol{\textrm{curl}}_h(\boldsymbol{v}_h \times \boldsymbol{\Theta }_h) - \boldsymbol{p}_h\Vert ^2_E \, . $$

Using Proposition 4, we obtain

$$ \begin{aligned} (\boldsymbol{p}_h, \boldsymbol{H}_h)&\ge \frac{1}{2}\gamma ^{-1} \sum _{E \in \Omega _h} \left( h_E^2\Vert \boldsymbol{\textrm{curl}}_h(\boldsymbol{v}_h \times \boldsymbol{\Theta }_h) \Vert ^2_E - C h_E^3 \Vert [\![\,\boldsymbol{\textrm{curl}}_h(\boldsymbol{v}_h \times \boldsymbol{\Theta }_h)\,]\!] \Vert ^2_{\partial E} \right) \\&\ge \frac{1}{2}\left| v_h\right| _\mathrm{\boldsymbol{\textrm{curl}}}^2 - C \gamma ^{-1} \mu _{J_2}^{-1} h \left| \boldsymbol{v}_h\right| _\textrm{cip}^2 \ge \frac{1}{2}\left| v_h\right| _\mathrm{\boldsymbol{\textrm{curl}}}^2 - C \mu _{J_2}^{-1} \left| \boldsymbol{v}_h\right| _\textrm{cip}^2 \, , \end{aligned} $$

where in the last inequality we simply used that $\gamma ^{-1} h \le 1$ by the definition of $\gamma$.

Using Cauchy-Schwarz inequality, Proposition 4, the definition of the jump operator, trace inequality, and (23), we have that

$$ \begin{aligned} \sum _{E \in \Omega _h} (\boldsymbol{H}_h \times \boldsymbol{n}_E , \boldsymbol{\Theta }\times \boldsymbol{v}_h)_{\partial E}&\gtrsim - \left( \mu _{J_1}^{-1} \sum _{f \in \Sigma _h} \Vert \boldsymbol{H}_h \Vert ^2_f \right) ^{\frac{1}{2}} \left( \mu _{J_1}\sum _{f \in \Sigma _h} \Vert [\![\,\boldsymbol{v}_h \times \boldsymbol{\Theta }\,]\!] \Vert ^2_f \right) ^{\frac{1}{2}} \\&\gtrsim - \left( \mu _{J_1}^{-1}\sum _{E \in \Omega _h} h_E^{-1}\Vert \boldsymbol{H}_h \Vert ^2_E \right) ^{\frac{1}{2}} \left| \boldsymbol{v}_h\right| _\textrm{cip} \\&\gtrsim - \gamma ^{-\frac{1}{2}}\left( \mu _{J_1}^{-1}\sum _{E \in \Omega _h} h_E^{-2}\Vert {\hat{\boldsymbol{H}}}_h \Vert ^2_E \right) ^{\frac{1}{2}} \left| \boldsymbol{v}_h\right| _\textrm{cip} \\&\gtrsim - \mu _{J_1}^{-\frac{1}{2}} \left| \boldsymbol{v}_h\right| _\textrm{cip} \left| \boldsymbol{v}_h\right| _\mathrm{\boldsymbol{\textrm{curl}}}\, . \end{aligned} $$

Gathering all the above estimates, we have obtained

30 $$\begin{aligned} \begin{aligned} \mathcal {A}_\textrm{stab}(\boldsymbol{v}_h, \boldsymbol{B}_h, 0, \boldsymbol{H}_h)&\ge {\tilde{C}}_1\left| \boldsymbol{v}_h\right| _\mathrm{\boldsymbol{\textrm{curl}}}^2 - {\tilde{C}}_2\mu _{J_2}^{-1} \left| \boldsymbol{v}_h\right| _\textrm{cip}^2 \\&\qquad - {\tilde{C}}_3\Big ((1 + \sigma _\textrm{M}h) \left\| \boldsymbol{B}_h\right\| _\textrm{M}^2 + \frac{| \boldsymbol{\Theta }|^2_{\boldsymbol{W}^1_\infty (\Omega )} \, h}{\sigma _\textrm{S}} \Vert \boldsymbol{v}_h \Vert ^2_S \\&\qquad + \Big (\frac{\mu _{J_1}+ \mu _{J_2}}{\mu _{J_1}\,\mu _{J_2}} \Big ) \left| \boldsymbol{v}_h\right| _\textrm{cip}^2\Big )^\frac{1}{2}\left| v_h\right| _\mathrm{\boldsymbol{\textrm{curl}}}^2 \, , \end{aligned} \end{aligned}$$

where the constants ${\tilde{C}}_i$ do not depend on the mesh size *h*. The thesis now follows by using Young inequality. $\square$

In the following lemma, we prove that $\left\| \boldsymbol{H}_h\right\| _\textrm{M}$ is controlled by the norms of $\boldsymbol{v}_h$.

#### Lemma 14

(Continuity of the norm) Let the mesh assumptions **(MA1)**, and **(MA2)** if $k=1$. Assume that the consistency assumption **(RA1)** holds. Then the test function $\boldsymbol{H}_h$ introduced in Proposition 13 satisfies

$$ \left\| \boldsymbol{H}_h\right\| _\textrm{M}^2 \lesssim \dfrac{\sigma _\textrm{M}}{\sigma _\textrm{S}} \Vert \boldsymbol{\Theta }\Vert ^2_{\boldsymbol{L}^\infty ({\Omega })} \left\| \boldsymbol{v}_h\right\| _\textrm{S}^2 + \left| \boldsymbol{v}_h\right| _\mathrm{\boldsymbol{\textrm{curl}}}^2 \,. $$

#### Proof

We need to estimate the two terms that appear in the definition of $\left\| \boldsymbol{H}_h\right\| _\textrm{M}$. For the first one, we have

$$ \begin{aligned} \sigma _\textrm{M}\Vert \boldsymbol{H}_h\Vert ^2&= \sigma _\textrm{M}\, \gamma ^{-2} \, \Vert {\hat{\boldsymbol{H}}}_h\Vert ^2 \le \sigma _\textrm{M}\sum _{E} h_E^{-2} \Vert {\hat{\boldsymbol{H}}}_h\Vert ^2_E \\&\lesssim \sigma _\textrm{M}\sum _{E} h_E^{2} \Vert \boldsymbol{p}_h\Vert ^2_E \lesssim \sigma _\textrm{M}\sum _{E} h_E^{2} \Vert \boldsymbol{\textrm{curl}}_h(\boldsymbol{v}_h \times \boldsymbol{\Theta }_h)\Vert ^2_E \\&\lesssim \sigma _\textrm{M}\Vert \boldsymbol{\Theta }\Vert _{\boldsymbol{L}^\infty ({\Omega })}^2 \Vert \boldsymbol{v}_h\Vert ^2 \lesssim \dfrac{\sigma _\textrm{M}}{\sigma _\textrm{S}} \Vert \boldsymbol{\Theta }\Vert ^2_{\boldsymbol{L}^\infty ({\Omega })} \left\| \boldsymbol{v}_h\right\| _\textrm{S}^2 \, . \end{aligned} $$

For the second term, we use an inverse polynomial inequality, property (23), and the definition of $\gamma$

$$ \begin{aligned} \nu _\textrm{M}\Vert \boldsymbol{\textrm{curl}}(\boldsymbol{H}_h)\Vert ^2&\lesssim \nu _\textrm{M}\sum _{E \in \Omega _h} h_E^{-2} \Vert \boldsymbol{H}_h \Vert ^2 = \nu _\textrm{M}\, \gamma ^{-2}\,\sum _{E \in \Omega _h} h_E^{-2} \Vert {\hat{\boldsymbol{H}}}_h \Vert ^2 \\&\lesssim \gamma ^{-1}\,\sum _{E \in \Omega _h} h_E^{2} \Vert \boldsymbol{\textrm{curl}}_h(\boldsymbol{v}_h \times \boldsymbol{\Theta }_h) \Vert ^2_E = \left| \boldsymbol{v}_h\right| _\mathrm{\boldsymbol{\textrm{curl}}}^2 \,. \end{aligned} $$

$$\square $$

Finally, we conclude this section by establishing the inf-sup condition for our method.

#### Theorem 15

Let the mesh assumptions **(MA1)**, **(MA2)** if $k=1$. Assume that the consistency assumption **(RA1)** holds. We have that:

31 $$\begin{aligned} \left\| \boldsymbol{v}_h\right\| _\textrm{stab} + \left\| \boldsymbol{B}_h\right\| _\textrm{M} \lesssim \sup _{(\boldsymbol{w}_h,\textbf{K}_h) \in \boldsymbol{Z}^h_k\times \boldsymbol{W}^h_k} \dfrac{\mathcal {A}_\textrm{stab}(\boldsymbol{v}_h, \boldsymbol{B}_h, \boldsymbol{w}_h, \textbf{K}_h)}{\left\| \boldsymbol{w}_h\right\| _\textrm{stab} + \left\| \textbf{K}_h\right\| _\textrm{M}} \qquad \forall (\boldsymbol{v}_h,\boldsymbol{B}_h) \in \boldsymbol{Z}^h_k\times \boldsymbol{W}^h_k. \end{aligned}$$

#### Proof

The proof becomes standard in light of the previous derivations. It is sufficient to combine Propositions 12 and 13 taking $(\boldsymbol{w}_h,\textbf{K}_h) = \kappa (\boldsymbol{v}_h,\boldsymbol{B}_h) + (0,\boldsymbol{H}_h)$ for a sufficiently large $\kappa$, in addition to recalling Lemma 14. Here, $\boldsymbol{H}_h$ is defined as in Proposition 13. $\square$

### Error Analysis

Let $(\boldsymbol{u}, p, \boldsymbol{B})$ and $(\boldsymbol{u}_h, p_h, \boldsymbol{B}_h)$ be the solutions of (9) and (13) respectively. We introduce the following notations that will be useful for the error analysis

32 $$\begin{aligned} \boldsymbol{e}_{\mathcal {I}}:= \boldsymbol{u}- \mathcal {I}_{\boldsymbol{V}}\boldsymbol{u}\,, \qquad \boldsymbol{e}_h:= \boldsymbol{u}_h - \mathcal {I}_{\boldsymbol{V}}\boldsymbol{u}\,, \qquad \boldsymbol{E}_{\mathcal {I}}:= \boldsymbol{B}- \mathcal {I}_{\boldsymbol{W}}\boldsymbol{B}\,, \qquad \boldsymbol{E}_h:= \boldsymbol{B}_h - \mathcal {I}_{\boldsymbol{W}}\boldsymbol{B}\,, \end{aligned}$$

where $\mathcal {I}_{\boldsymbol{V}}\boldsymbol{u}$ and $\mathcal {I}_{\boldsymbol{W}}\boldsymbol{B}$ are defined in Lemmas 5 and 6. We define also the following quantity

33 $$\begin{aligned} \begin{aligned} \Lambda _\textrm{S}^2&:= \max \biggl \{ \sigma _\textrm{S}h^2 \,, \max _{f \in \Sigma _h^\mathrm{{int}}} \Vert \boldsymbol{\chi }\cdot \boldsymbol{n}_f \Vert _{\boldsymbol{L}^\infty (f)} h \,, \Vert \boldsymbol{\Theta }\Vert _{\boldsymbol{W}^1_\infty ({\Omega })}^2 h \,, \nu _\textrm{S}\biggr \} \,. \end{aligned} \end{aligned}$$

We begin the error analysis with the following proposition.

#### Proposition 16

Let $(\boldsymbol{u}, \boldsymbol{B}) \in \boldsymbol{Z}\times \boldsymbol{W}$ and $(\boldsymbol{u}_h, \boldsymbol{B}_h) \in \boldsymbol{Z}^h_k\times \boldsymbol{W}^h_k$ be the solutions of (9) and (13) respectively. It holds that

$$ \left\| \boldsymbol{u}- \boldsymbol{u}_h\right\| _\textrm{stab} + \left\| \boldsymbol{B}- \boldsymbol{B}_h\right\| _\textrm{M} \lesssim \Xi _\mathcal {I} + \Xi _h \, , $$

where the two error quantities are defined as

$$ \Xi _{{\mathcal {I}}} := \left\| \boldsymbol{e}_{\mathcal {I}}\right\| _\textrm{stab} + \left\| \boldsymbol{E}_{\mathcal {I}}\right\| _\textrm{M} \, , $$

and

$$ \Xi _h := \sup _{(\boldsymbol{w}_h,\textbf{K}_h) \in \boldsymbol{Z}^h_k\times \boldsymbol{W}^h_k} \dfrac{\mathcal {A}_\textrm{stab}(\boldsymbol{u}, \boldsymbol{B}, \boldsymbol{w}_h, \textbf{K}_h) - \mathcal {A}_\textrm{stab}(\mathcal {I}_{\boldsymbol{V}}\boldsymbol{u}, \mathcal {I}_{\boldsymbol{W}}\boldsymbol{B}, \boldsymbol{w}_h, \textbf{K}_h) }{\left\| \boldsymbol{w}_h\right\| _\textrm{stab} + \left\| \textbf{K}_h\right\| _\textrm{M}} \, . $$

#### Proof

Using triangular inequality, we have that

$$ \left\| \boldsymbol{u}- \boldsymbol{u}_h\right\| _\textrm{stab} + \left\| \boldsymbol{B}- \boldsymbol{B}_h\right\| _\textrm{M} \le \left\| \boldsymbol{e}_{\mathcal {I}}\right\| _\textrm{stab} + \left\| \boldsymbol{E}_{\mathcal {I}}\right\| _\textrm{M} + \left\| \boldsymbol{e}_h\right\| _\textrm{stab} + \left\| \boldsymbol{E}_h\right\| _\textrm{M} \, . $$

Applying Theorem 15 and the consistency property (18), we obtain

$$ \begin{aligned} \left\| \boldsymbol{e}_h\right\| _\textrm{stab} + \left\| \boldsymbol{E}_h\right\| _\textrm{M}&\lesssim \sup _{(\boldsymbol{w}_h,\textbf{K}_h) \in \boldsymbol{Z}^h_k\times \boldsymbol{W}^h_k} \dfrac{\mathcal {A}_\textrm{stab}(\boldsymbol{e}_h, \boldsymbol{E}_h, \boldsymbol{w}_h, \textbf{K}_h)}{\left\| \boldsymbol{w}_h\right\| _\textrm{stab} + \left\| \textbf{K}_h\right\| _\textrm{M}} \\&\lesssim \sup _{(\boldsymbol{w}_h,\textbf{K}_h) \in \boldsymbol{Z}^h_k\times \boldsymbol{W}^h_k} \dfrac{\mathcal {A}_\textrm{stab}(\boldsymbol{u}_h, \boldsymbol{B}_h, \boldsymbol{w}_h, \textbf{K}_h) - \mathcal {A}_\textrm{stab}(\mathcal {I}_{\boldsymbol{V}}\boldsymbol{u}, \mathcal {I}_{\boldsymbol{W}}\boldsymbol{B}, \boldsymbol{w}_h, \textbf{K}_h) }{\left\| \boldsymbol{w}_h\right\| _\textrm{stab} + \left\| \textbf{K}_h\right\| _\textrm{M}} \\&\lesssim \sup _{(\boldsymbol{w}_h,\textbf{K}_h) \in \boldsymbol{Z}^h_k\times \boldsymbol{W}^h_k} \dfrac{\mathcal {A}_\textrm{stab}(\boldsymbol{u}, \boldsymbol{B}, \boldsymbol{w}_h, \textbf{K}_h) - \mathcal {A}_\textrm{stab}(\mathcal {I}_{\boldsymbol{V}}\boldsymbol{u}, \mathcal {I}_{\boldsymbol{W}}\boldsymbol{B}, \boldsymbol{w}_h, \textbf{K}_h) }{\left\| \boldsymbol{w}_h\right\| _\textrm{stab} + \left\| \textbf{K}_h\right\| _\textrm{M}} \, . \end{aligned} $$

The proof is concluded by recalling the definitions of $\Xi _{\mathcal {I}}$ and $\Xi _h$. $\square$

To properly estimate the rate of convergence of our method, we make the following stronger assumptions. Note that we allow for magnetic fields not in $H^1(\Omega )$, since we are considering also non-convex lipschitz domains.

**(RA2) Regularity assumptions on the exact solution (error analysis):**

Assume that:

- the velocity field satisfies $\boldsymbol{u}\in \boldsymbol{H}^{s}(\Omega _h)$ for some $\frac{3}{2} < s\le k+1,$
- the magnetic field satisfies $\boldsymbol{B}\in \boldsymbol{H}^{r}(\boldsymbol{\textrm{curl}},\Omega _h)$ for some $\frac{1}{2} < r\le k+1.$

#### Lemma 17

(Interpolation error) Let Assumption **(MA1)** hold. Furthermore, if $k=1$ let also Assumption **(MA2)** hold. Then, under the regularity assumption **(RA2)**, it holds that

$$ \Xi _{\mathcal {I}}^2 \lesssim \Lambda _\textrm{S}^2 \, h^{2s-2} \vert \boldsymbol{u}\vert _{s, \Omega _h}^2 + \sigma _\textrm{M}h^{2r} |\boldsymbol{B}|^2_{\boldsymbol{H}^r(\boldsymbol{\textrm{curl}},\Omega _h)} + \nu _\textrm{S}h^{2{\tilde{r}}}|\boldsymbol{\textrm{curl}}(\boldsymbol{B})|^2_{{\tilde{r}},\Omega _h}\, , $$

where ${\tilde{r}}:= \min \{ r,k \}$, see Lemma 6.

#### Proof

We recall that

$$ \Xi _{\mathcal {I}} = \left\| \boldsymbol{e}_{\mathcal {I}}\right\| _\textrm{stab} + \left\| \boldsymbol{E}_{\mathcal {I}}\right\| _\textrm{M} \, . $$

Exploiting the definition of $\left\| \cdot \right\| _\textrm{stab}$ and following the same steps of [6], we have that

34 $$\begin{aligned} \left\| \boldsymbol{e}_{\mathcal {I}}\right\| _\textrm{S}^2&\lesssim (\sigma _\textrm{S}h^2 + \nu _\textrm{S}(1 + \mu _a)) h^{2s-2} \vert \boldsymbol{u}\vert _{s, \Omega _h}^2 \lesssim \Lambda _\textrm{S}^2 h^{2s-2} \vert \boldsymbol{u}\vert _{s, \Omega _h}^2\,, \end{aligned}$$

35 $$\begin{aligned} \left| \boldsymbol{e}_{\mathcal {I}}\right| _\textrm{upw}^2&\lesssim \mu _c\max _{f \in \Sigma _h^\mathrm{{int}}} \Vert \boldsymbol{\chi }\cdot \boldsymbol{n}_f \Vert _{\boldsymbol{L}^\infty (f)} h^{2s-1} \vert \boldsymbol{u}\vert _{s, \Omega _h}^2 \lesssim \Lambda _\textrm{S}^2 h^{2s-2} \vert \boldsymbol{u}\vert _{s, \Omega _h}^2\, . \end{aligned}$$

For the jump term in the norm, we have that

$$ \left| \boldsymbol{e}_{\mathcal {I}}\right| _\textrm{cip}^2 = \mu _{J_1}\sum _{f \in \Sigma _h} \Vert [\![\,\boldsymbol{\Theta }\times \boldsymbol{e}_{\mathcal {I}}\,]\!]_f \Vert _{f}^2 + \mu _{J_2}\sum _{f \in \Sigma _h^\mathrm{{int}}} h_f^2 \Vert [\![\,\boldsymbol{\textrm{curl}}_h(\boldsymbol{e}_{\mathcal {I}}\times \boldsymbol{\Theta }_h)\,]\!]_f \Vert _{f}^2 \, . $$

For the first term, using trace inequality and Lemma 5, we obtain that

36 $$\begin{aligned} \begin{aligned} \mu _{J_1}\sum _{f \in \Sigma _h} \Vert [\![\,\boldsymbol{\Theta }\times \boldsymbol{e}_{\mathcal {I}}\,]\!]_f \Vert ^2_f&\lesssim \mu _{J_1}\sum _{f \in \Sigma _h} \Vert \boldsymbol{\Theta }\Vert _{\boldsymbol{L}^\infty ({f})}^2 \Vert \boldsymbol{e}_{\mathcal {I}}\Vert ^2_f \\&\lesssim \mu _{J_1}\sum _{f \in \Sigma _h} \Vert \boldsymbol{\Theta }\Vert _{\boldsymbol{L}^\infty ({f})}^2 \bigl (h_E^{-1}\Vert \boldsymbol{e}_{\mathcal {I}}\Vert ^2_E + h_E| \boldsymbol{e}_{\mathcal {I}}|^2_{1,E} \bigr ) \\&\lesssim \mu _{J_1}\max _{f \in \Sigma _h}\Vert \boldsymbol{\Theta }\Vert _{\boldsymbol{L}^\infty ({f})}^2 h^{2s-1} | \boldsymbol{u}|^2_{s,\Omega _h} \lesssim \Lambda _\textrm{S}^2 h^{2s-2} | \boldsymbol{u}|^2_{s,\Omega _h}\, . \end{aligned} \end{aligned}$$

Similarly, on the other term, using (29) together with the fact that $\boldsymbol{\Theta }_h$ is piecewise constant and $\textrm{div}(\boldsymbol{e}_{\mathcal {I}})=0$, we obtain

37 $$\begin{aligned} \begin{aligned} \mu _{J_2}\sum _{f \in \Sigma _h^\mathrm{{int}}} h_f^2 \Vert [\![\,\boldsymbol{\textrm{curl}}_h (\boldsymbol{e}_{\mathcal {I}}\times \boldsymbol{\Theta }_h)\,]\!]_f\Vert ^2&\lesssim \mu _{J_2}\sum _{f \in \Sigma _h^\mathrm{{int}}} h_f^2 \Vert \boldsymbol{\Theta }_h \Vert ^2_{\boldsymbol{L}^{\infty }(f^\pm )} \Vert \nabla \boldsymbol{e}_{\mathcal {I}}\Vert ^2_{\boldsymbol{L}^2(f^\pm )}\\  &\lesssim \Lambda _\textrm{S}^2 h^{2s-2}| \boldsymbol{u}|^2_{s,\Omega _h} \, . \end{aligned} \end{aligned}$$

Combining (36) and (37), we have

$$ \left| \boldsymbol{e}_{\mathcal {I}}\right| _\textrm{cip}^2 \lesssim \Lambda _\textrm{S}^2 h^{2s-2} | \boldsymbol{u}|_{s,\Omega _h}^2 \, . $$

For $\left| \boldsymbol{e}_{\mathcal {I}}\right| _\mathrm{\boldsymbol{\textrm{curl}}}$, recalling (29), we have that

$$ \begin{aligned} \left| \boldsymbol{e}_{\mathcal {I}}\right| _\mathrm{\boldsymbol{\textrm{curl}}}^2&= \gamma ^{-1} \, \sum _{E \in \Omega _h} h_E^2 \, \Vert \boldsymbol{\textrm{curl}}_h(\boldsymbol{e}_{\mathcal {I}}\times \boldsymbol{\Theta }_h) \Vert ^2_{E} \lesssim \gamma ^{-1} \, \sum _{E \in \Omega _h} h_E^2 \Vert \boldsymbol{\Theta }\Vert _{L^{\infty }({\Omega })}^2 \Vert \nabla \boldsymbol{e}_{\mathcal {I}}\Vert ^2_{E}\\&\lesssim h^{2s-1} \Vert \boldsymbol{\Theta }\Vert ^2_{\boldsymbol{L}^\infty ({\Omega })} | \boldsymbol{u}|^2_{s,\Omega _h} \lesssim \Lambda _\textrm{S}^2 h^{2s-2} |\boldsymbol{u}|_{s,\Omega _h}^2 \, . \end{aligned} $$

For the magnetic field, we have to use the interpolation estimate of Lemma 6. It holds that

$$ \begin{aligned} \left\| \boldsymbol{E}_{\mathcal {I}}\right\| _\textrm{M}^2&\lesssim \sum _{E \in \Omega _h} \sigma _\textrm{M}h^{2r}_E |\boldsymbol{B}|^2_{\boldsymbol{H}^r(\boldsymbol{\textrm{curl}},E)} + \sum _{E \in \Omega _h}\nu _\textrm{M}h_E^{2{\tilde{r}}}|\boldsymbol{\textrm{curl}}(\boldsymbol{B})|^2_{{\tilde{r}},E} \\&\lesssim \sigma _\textrm{M}h^{2r} |\boldsymbol{B}|^2_{\boldsymbol{H}^r(\boldsymbol{\textrm{curl}},\Omega _h)} + \nu _\textrm{M}h^{2{\tilde{r}}}|\boldsymbol{\textrm{curl}}(\boldsymbol{B})|^2_{{\tilde{r}},\Omega _h}\, . \end{aligned} $$

$$\square $$

#### Lemma 18

Let Assumption **(MA1)** hold. Furthermore, if $k=1$ let also Assumption **(MA2)** hold. Then, under the regularity assumption **(RA2)**. It holds that

$$ \begin{aligned} \Xi _h^2&\lesssim (\Lambda _\textrm{S}^2 + \Gamma _\textrm{S}^2 + \Gamma _\textrm{M}^2) h^{2s-2} \vert \boldsymbol{u}\vert ^2_{s, \Omega _h} + \nu _\textrm{S}h^{2{\tilde{r}}}|\boldsymbol{\textrm{curl}}(\boldsymbol{B})|^2_{{\tilde{r}},\Omega _h} \\&\quad + (\sigma _\textrm{M}h^{2r} + (\Gamma _\textrm{S}^2+\Gamma _\textrm{M}^2 +1 ) h^{2r-1} ) \bigl |\boldsymbol{B}|_{\boldsymbol{H}^{r}(\boldsymbol{\textrm{curl}},\Omega _h)}^2 \, , \end{aligned} $$

where

38 $$\begin{aligned} \begin{aligned} \Gamma _\textrm{S}^2&:= \min \{ \sigma _\textrm{S}^{-1} h^2, \, \max \{\sigma _\textrm{S}^{-1},\nu _\textrm{S}^{-1}\}h^4\} \Vert \boldsymbol{\chi }\Vert _{\boldsymbol{W}^1_\infty ({\Omega })}^2 + \sigma _\textrm{S}^{-1} h\Vert \boldsymbol{\Theta }\Vert _{\boldsymbol{W}^1_\infty ({\Omega })}^2\, ,\\ \Gamma _\textrm{M}^2&:= \min \{ \sigma _\textrm{M}^{-1} h^2, \, \nu _\textrm{M}^{-1} h^4 \} \Vert \boldsymbol{\Theta }\Vert _{\boldsymbol{W}^1_\infty ({\Omega })}^2 + \gamma h^{-1} \,. \end{aligned} \end{aligned}$$

#### Proof

We recall that by definition

$$\begin{aligned}  &   \mathcal {A}_\textrm{stab}(\boldsymbol{u}, \boldsymbol{B}, \boldsymbol{w}_h, \textbf{K}_h) - \mathcal {A}_\textrm{stab}(\mathcal {I}_{\boldsymbol{V}}\boldsymbol{u}, \mathcal {I}_{\boldsymbol{W}}\boldsymbol{B}, \boldsymbol{w}_h, \textbf{K}_h)\\  &   \qquad = \bigl ( \sigma _\textrm{S}(\boldsymbol{e}_{\mathcal {I}}, \boldsymbol{w}_h) + \nu _\textrm{S}a^\textrm{S}_h(\boldsymbol{e}_{\mathcal {I}}, \boldsymbol{w}_h) \bigr )\\  &   \qquad \quad + \bigl (\sigma _\textrm{M}(\boldsymbol{E}_{\mathcal {I}}, \textbf{K}_h) + \nu _\textrm{M}a^\textrm{M}(\boldsymbol{E}_{\mathcal {I}}, \textbf{K}_h) \bigr ) \\  &   \qquad \quad + c_h(\boldsymbol{e}_{\mathcal {I}}, \boldsymbol{w}_h) -d(\boldsymbol{E}_{\mathcal {I}}, \boldsymbol{w}_h) + d(\textbf{K}_h, \boldsymbol{e}_{\mathcal {I}}) + J_h(\boldsymbol{e}_{\mathcal {I}}, \boldsymbol{w}_h) =: \sum _{i=1}^6 \alpha _i \,. \end{aligned}$$

We need to estimate each of these six terms. Some of these terms are estimated similarly to [6], so we only state the result.

$\bullet$ Estimate of $\alpha _1$ and $\alpha _3$: using the same calculations of [6], we have that

39 $$\begin{aligned} \alpha _1 \lesssim \Lambda _\textrm{S}\, h^{s-1} \vert \boldsymbol{u}\vert _{s, \Omega _h} \left\| \boldsymbol{w}_h\right\| _\textrm{stab} \, , \qquad \alpha _3 \lesssim (\Lambda _\textrm{S}+ \Gamma _\textrm{S}) h^{s-1} \vert \boldsymbol{u}\vert _{s, \Omega _h} \left\| \boldsymbol{w}_h\right\| _\textrm{stab} \,. \end{aligned}$$

$\bullet$ Estimate of $\alpha _2$: Using the interpolation estimate of Lemma 6, we have that

40 $$\begin{aligned} \begin{aligned} \bigl (\sigma _\textrm{M}(\boldsymbol{E}_{\mathcal {I}}, \textbf{K}_h) + \nu _\textrm{M}a^\textrm{M}(\boldsymbol{E}_{\mathcal {I}}, \textbf{K}_h) \bigr )&\le \sigma _\textrm{M}\Vert \boldsymbol{E}_{\mathcal {I}}\Vert \Vert \textbf{K}_h\Vert + \nu _\textrm{M}\Vert \boldsymbol{\textrm{curl}}(\boldsymbol{E}_{\mathcal {I}}) \Vert \, \Vert \boldsymbol{\textrm{curl}}(\textbf{K}_h)\Vert \\&\lesssim \bigl (\sigma _\textrm{M}^\frac{1}{2} h^{r} \bigl |\boldsymbol{B}|_{\boldsymbol{H}^{r}(\boldsymbol{\textrm{curl}},\Omega _h)} + \nu _\textrm{M}^\frac{1}{2} h^{{\tilde{r}}} |\boldsymbol{\textrm{curl}}(\boldsymbol{B})|_{{\tilde{r}},\Omega _h} \bigr )\left\| \textbf{K}_h\right\| _\textrm{M} \, . \end{aligned} \end{aligned}$$

$\bullet$ Estimate of $\alpha _4$: Following [6], it holds that by integration by parts

41 $$\begin{aligned} \begin{aligned} \alpha _4&= (\boldsymbol{E}_{\mathcal {I}}, \boldsymbol{\textrm{curl}}_h (\boldsymbol{w}_h \times (\boldsymbol{\Theta }- \boldsymbol{\Theta }_h))) + (\boldsymbol{E}_{\mathcal {I}}, \boldsymbol{\textrm{curl}}_h (\boldsymbol{w}_h \times \boldsymbol{\Theta }_h)) + \\&\quad + \sum _{f \in \Sigma _h} (\boldsymbol{E}_{\mathcal {I}}\times \boldsymbol{n}_f , [\![\,\boldsymbol{w}_h \times \boldsymbol{\Theta }\,]\!])_f =: \sum _{j=1}^3 \alpha _{4,j} \, . \end{aligned} \end{aligned}$$

For the first term, mimicking the steps of [6] we have that

42 $$\begin{aligned} \begin{aligned} \alpha _{4,1} \lesssim \Gamma _\textrm{S}h^{r-\frac{1}{2}} \vert \boldsymbol{B}\vert _{\boldsymbol{H}^{r}(\boldsymbol{\textrm{curl}},\Omega _h)} \left\| \boldsymbol{w}_h\right\| _\textrm{S} \, . \end{aligned} \end{aligned}$$

Thanks to the definition of $\left| \cdot \right| _\mathrm{\boldsymbol{\textrm{curl}}}$, we obtain

$$ \begin{aligned} \alpha _{4,2} = ( \boldsymbol{E}_{\mathcal {I}}\,, \boldsymbol{\textrm{curl}}_h (\boldsymbol{w}_h \times \boldsymbol{\Theta }_h) )&\lesssim \left( \sum _{E \in \Omega _h} h_E^{-2} \Vert \boldsymbol{E}_{\mathcal {I}}\Vert ^2_E\right) ^\frac{1}{2} \left( \sum _{E \in \Omega _h} h_E^{2} \Vert \boldsymbol{\textrm{curl}}_h (\boldsymbol{w}_h \times \boldsymbol{\Theta }_h)\Vert ^2_E\right) ^\frac{1}{2} \\&\lesssim \gamma ^\frac{1}{2} \left( \sum _{E \in \Omega _h} h_E^{-2} \Vert \boldsymbol{E}_{\mathcal {I}}\Vert ^2_E\right) ^\frac{1}{2} \left| \boldsymbol{w}_h\right| _\mathrm{\boldsymbol{\textrm{curl}}} \\&\lesssim \gamma ^{\frac{1}{2}} h^{r-1} | \boldsymbol{B}|_{\boldsymbol{H}^r(\boldsymbol{\textrm{curl}},\Omega _h)}\left| \boldsymbol{w}_h\right| _\mathrm{\boldsymbol{\textrm{curl}}} \\&\lesssim {\Gamma _\textrm{M}} h^{r-\frac{1}{2}} | \boldsymbol{B}|_{\boldsymbol{H}^{r}(\boldsymbol{\textrm{curl}},\Omega _h)} \left| \boldsymbol{w}_h\right| _\mathrm{\boldsymbol{\textrm{curl}}} \, . \end{aligned} $$

Finally,

43 $$\begin{aligned} \begin{aligned} \alpha _{4,3}&\le \biggl (\mu _{J_1}^{-1} \sum _{f \in \Sigma _h} \Vert \boldsymbol{E}_{\mathcal {I}}\Vert _{f}^2 \biggr )^{1/2} \left| \boldsymbol{w}_h\right| _\textrm{cip}\\  &\lesssim \biggl (\mu _{J_1}^{-1} \sum _{E \in \Omega _h} (h_E^{-1} \Vert \boldsymbol{E}_{\mathcal {I}}\Vert _{E}^2 + h_E^{2{\hat{r}}-1} \vert \boldsymbol{E}_{\mathcal {I}}\vert _{{\hat{r}},E}^2) \biggr )^{1/2} \left| \boldsymbol{w}_h\right| _\textrm{cip} \\&\lesssim h^{r-\frac{1}{2}} |\boldsymbol{B}|_{\boldsymbol{H}^r(\boldsymbol{\textrm{curl}},\Omega _h)} \left| \boldsymbol{w}_h\right| _\textrm{cip} \lesssim h^{r-\frac{1}{2}} \vert \boldsymbol{B}\vert _{\boldsymbol{H}^r(\boldsymbol{\textrm{curl}},\Omega _h)} \left| \boldsymbol{w}_h\right| _\textrm{cip} \end{aligned} \end{aligned}$$

where $${\hat{r}}:= \min \{r,1\}$$.

$\bullet$ Estimate of $\alpha _5$: Exploiting the orthogonality property of the interpolation operator, and standard approximation results, we obtain

44 $$\begin{aligned} \begin{aligned} \alpha _5&= (\boldsymbol{\textrm{curl}}({\textbf{K}}_h) \times \boldsymbol{\Theta }\,, \boldsymbol{e}_{\mathcal {I}}) = (\boldsymbol{\textrm{curl}}({\textbf{K}}_h) \times (\boldsymbol{\Theta }-\boldsymbol{\Theta }_h) \,, \boldsymbol{e}_{\mathcal {I}}) \\&\le \sum _{E \in \Omega _h} \Vert \boldsymbol{\Theta }- \boldsymbol{\Theta }_h \Vert _{\boldsymbol{L}^\infty (E)} \Vert \boldsymbol{\textrm{curl}}({\textbf{K}}_h) \Vert _{E} \Vert \boldsymbol{e}_{\mathcal {I}}\Vert _{E} \\&\lesssim \sum _{E \in \Omega _h} h_E \Vert \boldsymbol{\Theta }\Vert _{\boldsymbol{W}^1_\infty (E)} \Vert \boldsymbol{\textrm{curl}}({\textbf{K}}_h) \Vert _{E} \Vert \boldsymbol{e}_{\mathcal {I}}\Vert _{E} \\&\lesssim \Vert \boldsymbol{\Theta }\Vert _{\boldsymbol{W}^1_\infty ({\Omega })} \Vert \boldsymbol{e}_{\mathcal {I}}\Vert \biggl (\sum _{E \in \Omega _h} h_E^2 \Vert \boldsymbol{\textrm{curl}}({\textbf{K}}_h) \Vert _{E}^2 \biggr )^{1/2} \\&\lesssim \min \{ \sigma _\textrm{M}^{-1/2}, \nu _\textrm{M}^{-1/2} h\} \Vert \boldsymbol{\Theta }\Vert _{\boldsymbol{W}^1_\infty ({\Omega })} h^{s} \vert \boldsymbol{u}\vert _{s, \Omega _h} \left\| {\textbf{K}}_h\right\| _\textrm{M} \\&\lesssim \Gamma _\textrm{M}h^{s-1} \vert \boldsymbol{u}\vert _{s, \Omega _h} \left\| {\textbf{K}}_h\right\| _\textrm{M} \, . \end{aligned} \end{aligned}$$

$\bullet$ Estimate of $\alpha _6$: By definition, we have that

$$ \begin{aligned} \alpha _6&= \sum _{f \in \Sigma _h} \bigl ( \mu _{J_1}( [\![\,\boldsymbol{\Theta }\times \boldsymbol{e}_{\mathcal {I}}\,]\!]_f , [\![\,\boldsymbol{\Theta }\times \boldsymbol{w}_h\,]\!]_f \bigr )_f \\&\qquad + \mu _{J_2}h_f^2 \bigl ([\![\,\boldsymbol{\textrm{curl}}_h (\boldsymbol{e}_{\mathcal {I}}\times \boldsymbol{\Theta })\,]\!]_f ,[\![\,\boldsymbol{\textrm{curl}}_h (\boldsymbol{w}_h \times \boldsymbol{\Theta })\,]\!]_f \bigr )_f =: \sum _{j=1}^2 \alpha _{6,j} \, . \end{aligned} $$

On the first one, using (36), we have that

$$ \begin{aligned} \alpha _{6,1}&= \mu _{J_1}\sum _{f \in \Sigma _h} \bigl ( [\![\,\boldsymbol{\Theta }\times \boldsymbol{e}_{\mathcal {I}}\,]\!]_f , [\![\,\boldsymbol{\Theta }\times \boldsymbol{w}_h\,]\!]_f \bigr )_f \lesssim \left| \boldsymbol{e}_{\mathcal {I}}\right| _\textrm{cip} \left| \boldsymbol{w}_h\right| _\textrm{cip}\\  &\lesssim \Lambda _\textrm{S}h^{s-1} |\boldsymbol{u}|_{s,\Omega _h}\left\| \boldsymbol{w}_h\right\| _\textrm{stab} \, . \end{aligned} $$

Using the Cauchy-Schwarz and triangular inequalities, estimates in the same spirit of (21), and bound (37), we obtain (without showing all the details)

$$\begin{aligned} \alpha _{6,2}\le &   \mu _{J_2}\sum _{f \in \Sigma _h^\mathrm{{int}}} h_f^2 \bigl ([\![\,\boldsymbol{\textrm{curl}}_h (\boldsymbol{e}_{\mathcal {I}}\times \boldsymbol{\Theta })\,]\!]_f ,[\![\,\boldsymbol{\textrm{curl}}_h (\boldsymbol{w}_h \times \boldsymbol{\Theta })\,]\!]_f \bigr )_f \\\lesssim &   \left( \mu _{J_2}\sum _{f \in \Sigma _h^\mathrm{{int}}} h_f^2 \bigl (\Vert [\![\,\boldsymbol{\textrm{curl}}_h (\boldsymbol{e}_{\mathcal {I}}\times (\boldsymbol{\Theta }-\boldsymbol{\Theta }_h))\,]\!] \Vert ^2_f + \Vert [\![\,\boldsymbol{\textrm{curl}}_h (\boldsymbol{e}_{\mathcal {I}}\times \boldsymbol{\Theta }_h)\,]\!] \Vert ^2_f\bigr ) \right) ^{\frac{1}{2}}\left\| \boldsymbol{w}_h\right\| _\textrm{stab} \\\lesssim &   \left( \left| \boldsymbol{e}_{\mathcal {I}}\right| _\textrm{cip}^2 + \sum _{E \in \Omega _h} \Vert \boldsymbol{\Theta }\Vert _{\boldsymbol{W}^1_\infty (E)}^2 \big ( h_E \Vert \boldsymbol{e}_{\mathcal {I}}\Vert ^2_E + h_E^{2s+1} | \boldsymbol{e}_{\mathcal {I}}|^2_{s,E} \big ) \right) ^{\frac{1}{2}}\\  &   \left\| \boldsymbol{w}_h\right\| _\textrm{stab}\\\lesssim &   \Lambda _\textrm{S}h^{s-1} |\boldsymbol{u}|_{s,\Omega _h}\left\| \boldsymbol{w}_h\right\| _\textrm{stab} \, . \end{aligned}$$

Note that, differently from (21) and since $\boldsymbol{e}_{\mathcal {I}}$ is not a discrete function, here above we applied a scaled trace inequality from $\boldsymbol{H}^1(f)$ to $\boldsymbol{H}^s(E)$, with *f* face of element *E* (we recall $s>3/2$ by assumption), instead of an inverse inequality.

$$\square $$

Collecting the previous result we obtain the final estimate.

#### Theorem 19

Let Assumption **(MA1)** hold. Furthermore, if $k=1$ let also Assumption **(MA2)** be valid. Then, under the regularity assumption **(RA2)** it holds

$$ \begin{aligned} \left\| \boldsymbol{u}- \boldsymbol{u}_h\right\| _\textrm{stab}^2 + \left\| \boldsymbol{B}- \boldsymbol{B}_h\right\| _\textrm{M}^2&\lesssim (\Lambda _\textrm{S}^2 + \Gamma _\textrm{S}^2 + \Gamma _\textrm{M}^2) h^{2s-2} \vert \boldsymbol{u}\vert ^2_{s, \Omega _h} + \nu _\textrm{S}h^{2{\tilde{r}}}|\boldsymbol{\textrm{curl}}(\boldsymbol{B})|^2_{{\tilde{r}},\Omega _h} \\&\quad + (\sigma _\textrm{M}h^{2r} + (\Gamma _\textrm{S}^2+\Gamma _\textrm{M}^2+1 ) h^{2r-1} ) \bigl |\boldsymbol{B}|_{\boldsymbol{H}^{r}(\boldsymbol{\textrm{curl}},\Omega _h)}^2 \, . \end{aligned} $$

We now study the error on the pressure variable. We first recall that, exploiting the inf-sup condition for the $\boldsymbol{\textrm{BDM}}$ elements and the Poincarè inequality, there exists $\boldsymbol{w}_h \in \boldsymbol{V}^h_k$ such that

45 $$\begin{aligned} \Vert \boldsymbol{w}_h \Vert _{1,h} \lesssim 1 \,, \qquad \text {and} \qquad \Vert p_h - \Pi _{k-1} p\Vert \lesssim b(\boldsymbol{w}_h \,, p_h - \Pi _{k-1} p) \,. \end{aligned}$$

where the discrete norm is defined by:

$$ \Vert \boldsymbol{w}_h \Vert _{1,h}^2 := \sum _{E \in \Omega _h} \Vert \boldsymbol{w}_h \Vert _{1,E} + \sum _{f \in \Sigma _h} h_f^{-1} \Vert [\![\,\boldsymbol{w}_h\,]\!]_f \Vert _{f}^2 \, . $$

Furthermore, thank to the inclusion $\textrm{div}(\boldsymbol{V}^h_k) \subseteq {\mathbb {P}}_{k-1}(\Omega _h)$, it also holds

46 $$\begin{aligned} b(\boldsymbol{v}_h \,, p) = b(\boldsymbol{v}_h \,, \Pi _{k-1} p) \qquad \text {for all } \boldsymbol{v}_h \in \boldsymbol{V}^h_k. \end{aligned}$$

We are now ready to prove the following error estimates.

#### Theorem 20

(Error estimates for the pressure) Under the same assumptions as in Theorem 19, if $p \in H^s(\Omega )$ with $0 \le s \le k$, we have that

47 $$\begin{aligned} \begin{aligned} \Vert p - p_h \Vert&\lesssim (\Phi _\textrm{S}+ \Lambda _\textrm{S}+ \Vert \boldsymbol{\chi }\Vert _{\boldsymbol{L}^\infty (\Omega )} \sigma _\textrm{S}^{-1/2} ) \left\| \boldsymbol{u}- \boldsymbol{u}_h\right\| _\textrm{stab} + { h^s \, \vert p \vert _{s, \Omega _h}} \\&\quad + \Vert \boldsymbol{\Theta }\Vert _{\boldsymbol{W}^1_\infty ({\Omega })} \big ( \sigma _\textrm{M}^{-1/2}\left\| \boldsymbol{B}- \boldsymbol{B}_h\right\| _\textrm{M} + h^{r} \Vert \boldsymbol{B}\Vert _{\boldsymbol{H}^{r}(\boldsymbol{\textrm{curl}},\Omega )} \big ) \, . \end{aligned} \end{aligned}$$

where $$\Phi _\textrm{S}^2:= \max \{\nu _\textrm{S}, \sigma _\textrm{S}\}.$$

#### Proof

We only sketch the proof since it follows standard arguments in this type of problems and analogous steps with respect to [5]. We first recall that from Lemma 2 it holds

48 $$\begin{aligned} \Vert p - \Pi _{k-1} p \Vert \lesssim h^{k} | p |_{k,\Omega _h} \, . \end{aligned}$$

Exploiting estimates (45) and equation (46) in Problems (9) and (13), we obtain

49 $$\begin{aligned} \begin{aligned} \Vert p_h - \Pi _{k-1} p\Vert&\lesssim b(\boldsymbol{w}_h \,, p_h - \Pi _{k-1} p) = b(\boldsymbol{w}_h \,, p_h - p) \\&= \bigl ( \sigma _\textrm{S}(\boldsymbol{u}- \boldsymbol{u}_h, \boldsymbol{w}_h) + \nu _\textrm{S}a^\textrm{S}_h(\boldsymbol{u}- \boldsymbol{u}_h, \boldsymbol{w}_h) \bigr )+ c_h(\boldsymbol{u}- \boldsymbol{u}_h, \boldsymbol{w}_h) + \\&\quad -d(\boldsymbol{B}- \boldsymbol{B}_h, \boldsymbol{w}_h) + J_h(\boldsymbol{u}- \boldsymbol{u}_h, \boldsymbol{w}_h) =: \sum _{i=1}^4 \beta _i \,. \end{aligned} \end{aligned}$$

Following Lemma 18 and [6], we infer

50 $$\begin{aligned} \begin{aligned} \beta _1&\lesssim \Phi _\textrm{S}\left\| \boldsymbol{u}- \boldsymbol{u}_h\right\| _\textrm{stab} \,, \\ \beta _2&\lesssim (\Lambda _\textrm{S}+ \Vert \boldsymbol{\chi }\Vert _{\boldsymbol{L}^\infty (\Omega )} \sigma _\textrm{S}^{-1/2} )\left\| \boldsymbol{u}- \boldsymbol{u}_h\right\| _\textrm{stab} \,, \\ \beta _4&\lesssim \Lambda _\textrm{S}\left\| \boldsymbol{u}- \boldsymbol{u}_h\right\| _\textrm{stab} \, . \end{aligned} \end{aligned}$$

The term $\beta _3$ can be easily estimated, first integrating by parts to get

$$ \beta _3 = \sum _{E \in \Omega _h} (\boldsymbol{B}-\boldsymbol{B}_h, \, \boldsymbol{\textrm{curl}}(\boldsymbol{w}_h \times \boldsymbol{\Theta }))_E + \sum _{f \in \Sigma _h} ((\boldsymbol{B}-\boldsymbol{B}_h)\times \boldsymbol{n}_f, \, [\![\,\boldsymbol{w}_h\,]\!]_f \times \boldsymbol{\Theta })_f \, . $$

Afterwards, we bound the two terms above using Hölder and trace inequalities, recalling $\boldsymbol{\Theta }\in \boldsymbol{C}^1(\overline{\Omega })$ and $\Vert \boldsymbol{w}_h \Vert _{1,h} \lesssim 1$ (we avoid showing the details). We obtain

$$ \beta _3 \lesssim \Vert \boldsymbol{\Theta }\Vert _{\boldsymbol{W}^1_\infty ({\Omega })} \big ( \Vert \boldsymbol{B}- \boldsymbol{B}_h \Vert _{L^2(\Omega )} + h^{r} \Vert \boldsymbol{B}\Vert _{\boldsymbol{H}^{r}(\boldsymbol{\textrm{curl}},\Omega )} \big ) \, . $$

Estimate (47) now follows from (48), (49), (50) and the above bound for $\beta _3$.

$$\square $$

Gathering the results above and assuming maximum regularity of datum and solution, we have the following corollary stating the convergence rate of the method in a classical situation.

#### Corollary 21

Let Assumption **(MA1)** hold (as usual, if $k=1$ let also Assumption **(MA2)** be valid). Furthermore, suppose uniformly positive reaction terms $\sigma _\textrm{S}\sim \sigma _\textrm{M}\sim 1$ and that the regularity assumption **(RA2)** holds for $s=r=k+1$. Then we have

$$ \begin{aligned} \left\| \boldsymbol{u}- \boldsymbol{u}_h\right\| _\textrm{stab}^2 + \left\| \boldsymbol{B}- \boldsymbol{B}_h\right\| _\textrm{M}^2&\lesssim (h + \nu _\textrm{S}+ \nu _\textrm{M}) h^{2k} \, . \end{aligned} $$

In addition, if $p \in H^k(\Omega )$,

$$ \Vert p - p_h \Vert _{L^2(\Omega )}^2 \simeq \big (1 + (\nu _\textrm{S}+1)(h+\nu _\textrm{M}+\nu _\textrm{S})\big )h^{2k} $$.

For completeness, we close this section with the following convergence result without requirements on the analytical solution regularity.

#### Proposition 22

Let Assumption (MA1) hold. Let $\{\textbf{u}_h, p_h, \textbf{B}_h\}_h$ denote a sequence of solutions of the discrete problem (13) with mesh parameter *h* tending to zero, and let $(\textbf{u}, p, \textbf{B}) \in \boldsymbol{V}\times Q\times \boldsymbol{W}$ be the solution of the continuous problem (9). Then, for vanishing *h*, it holds (for any $2 \le p < 6$)

$$ \boldsymbol{u}_h \rightarrow \boldsymbol{u}\quad \text {in } L^p(\Omega ), \qquad p_h \rightharpoonup p \quad \text {weakly in } L^2(\Omega ) \, , $$

$$ \boldsymbol{B}_h \rightarrow \boldsymbol{B}\quad \text {in } L^2(\Omega ), \qquad \boldsymbol{B}_h \rightharpoonup \boldsymbol{B}\quad \text {weakly in } \boldsymbol{H}(\boldsymbol{\textrm{curl}}, \Omega ) \, . $$

#### Proof

The proof of this result very closely mimics the one of Proposition 5.4 in [14]. Thus, we only underline the main difference: in order to handle the convergence of the magnetic field $\boldsymbol{B}_h$, instead of using the compact inclusion of $\boldsymbol{H}^1(\Omega )$ into ${\boldsymbol{L}}^p(\Omega )$, $2 \le p < 6$, we use the discrete compactness result in [10, 11], which holds also thanks to Remark 9. $\square$

## Numerical Results

In this section, we develop a set of numerical tests for our method, with the aim of comparing the actual converge rates with the rates expected by the theory. Furthermore, we compare our approach with the one proposed in [6] in a non-convex domain. The proposed method has been implemented in C++, taking inspiration from the library Vem++ [15]. Readers interested in the code can contact the owner of the library to obtain access.

**Fig. 1:**
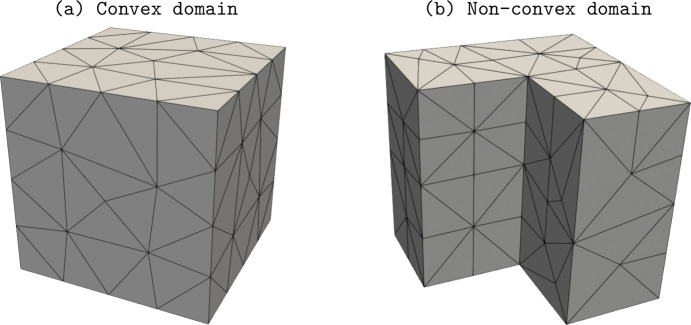
Example of meshes used for the tests (Unit cube and L-shaped domain)

In the first and second test we consider a cubic domain $\Omega = [0,1]^3$, in the third test an extruded L-shaped domain $\Omega = [-1,1]^3 \setminus ([-1,0)^2 \times [-1,1])$, while in the fourth test $\Omega = [0,10] \times [-2,2] \times [-1,1]$. In all cases we use a family of unstructured tetrahedral meshes of characteristic size *h*. Figure 1 illustrates two sample meshes, one for each domain.

We consider the following error quantities:

- $H^1(\Omega )$-seminorm of the velocity field;
- $H(\boldsymbol{\textrm{curl}},\Omega )$-seminorm of the magnetic field;
- $L^2(\Omega )$ norm of the pressure;
- a "total" norm given by
  $$ \begin{aligned} \Vert (\boldsymbol{u}-\boldsymbol{u}_h,\boldsymbol{B}-\boldsymbol{B}_h) \Vert _{\text {total}}&:= \sigma _\textrm{S}\Vert \boldsymbol{u}- \boldsymbol{u}_h \Vert + \nu _\textrm{S}\Vert \boldsymbol{\nabla }(\boldsymbol{u}- \boldsymbol{u}_h) \Vert \\&\quad +\sigma _\textrm{M}\Vert \boldsymbol{B}- \boldsymbol{B}_h \Vert + \nu _\textrm{M}\Vert \boldsymbol{\textrm{curl}}(\boldsymbol{B}- \boldsymbol{B}_h) \Vert \\&\quad + \mu _{J_1}\sum _{f \in \Sigma _h} \Vert [\![\,\boldsymbol{\Theta }\times (\boldsymbol{u}-\boldsymbol{u}_h)\,]\!]_f \Vert _{f}^2 \\&\quad + \mu _{J_2}\sum _{f \in \Sigma _h^\mathrm{{int}}} h_f^2 \Vert [\![\,\boldsymbol{\textrm{curl}}_h((\boldsymbol{u}-\boldsymbol{u}_h)\times \boldsymbol{\Theta })\,]\!]_f \Vert _{f}^2 \, . \end{aligned} $$

Test 1: Convergence study for a regular case. In this paragraph, we analyze the convergence of the error with respect to the parameter *h*. We consider the convex domain $\Omega = [0,1]^3$ and the polynomial degrees $k=1,2$. We design a problem for which the fields

$$ \boldsymbol{u}(x,y,z) := \begin{bmatrix} \sin (\pi x) \cos (\pi y) \cos (\pi z)\\ \cos (\pi x) \sin (\pi y) \cos (\pi z)\\ -2 \cos (\pi x) \cos (\pi y) \sin (\pi z) \end{bmatrix} , \qquad \boldsymbol{B}(x,y,z) := \begin{bmatrix} \sin (\pi y) \\ \sin (\pi z) \\ \sin (\pi x) \end{bmatrix}, $$

$$ p(x,y,z) = \sin (\pi x) + \sin (\pi y) - 2 \sin (\pi z) $$

are solutions of (9), where the functions $\boldsymbol{\chi }$ and $\boldsymbol{\Theta }$ are set to

$$ \boldsymbol{\chi }= \boldsymbol{u}, \qquad \boldsymbol{\Theta }= \boldsymbol{B}. $$

**Fig. 2:**
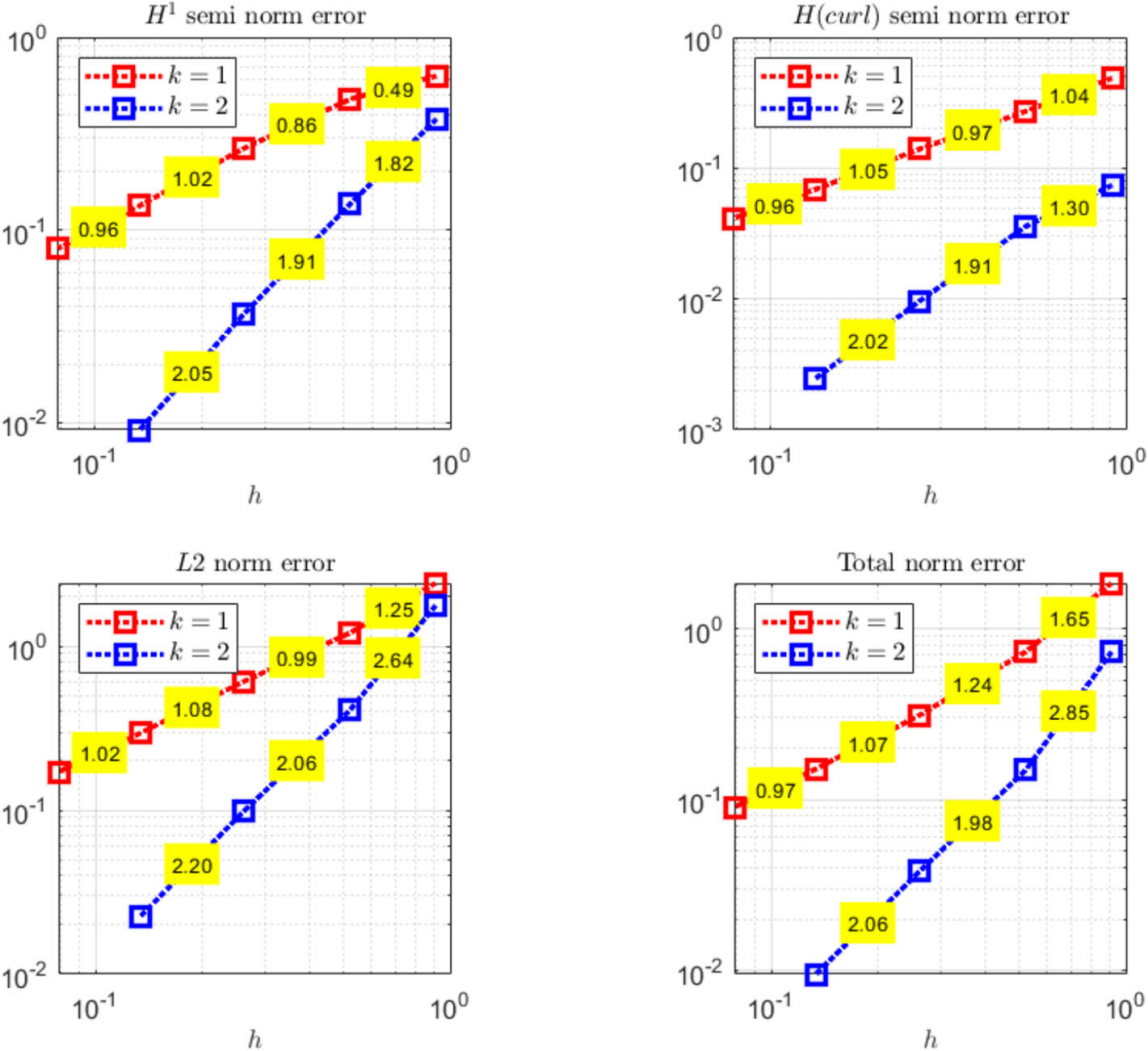
Numerical results for Test 1, diffusion dominated case ($\nu = 1$)

**Fig. 3:**
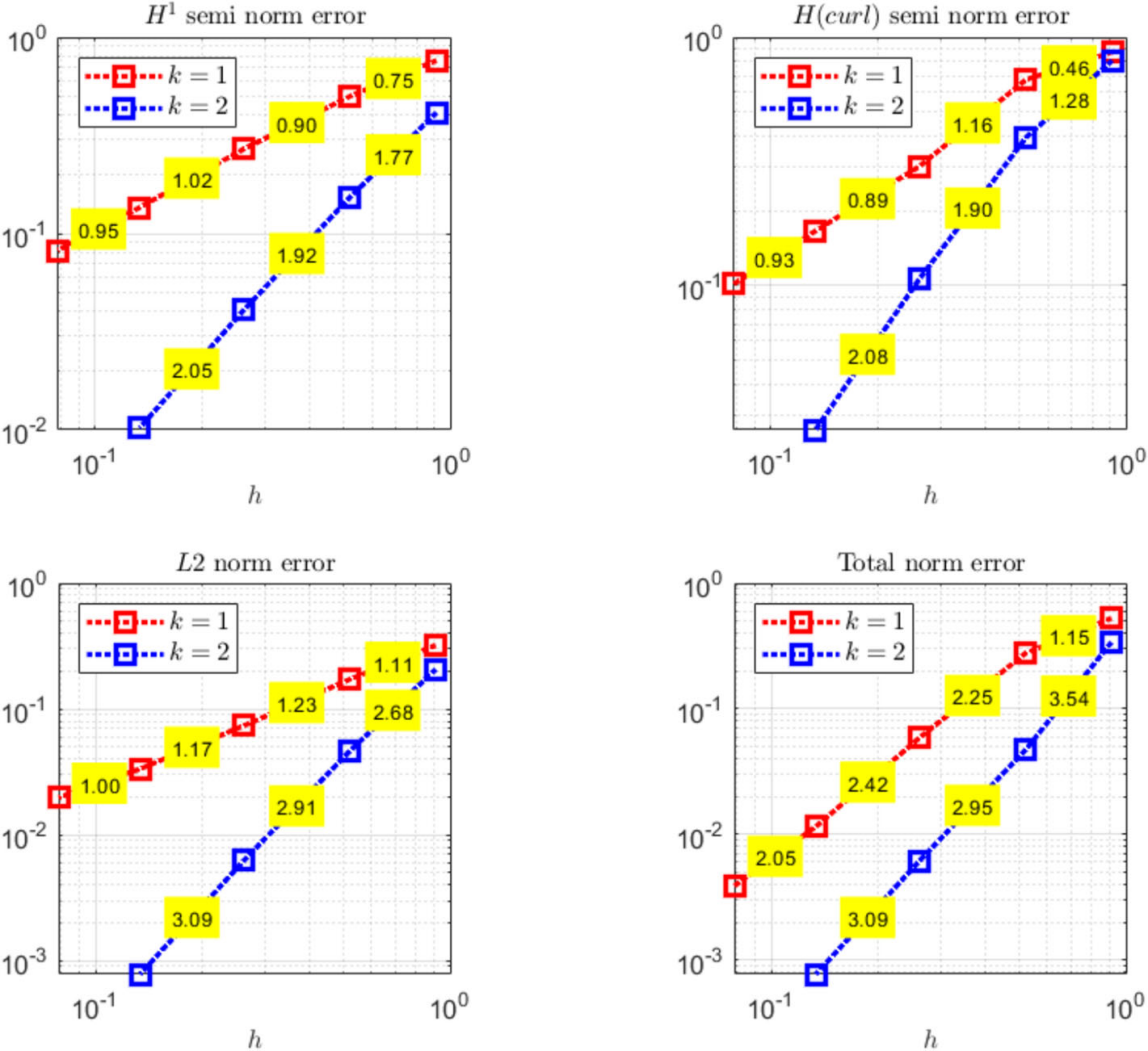
Numerical results for Test 1, convection dominated case ($\nu = 10^{-6}$)

Two choices for the parameters $\nu = \nu _\textrm{M}= \nu _\textrm{S}$ are considered, while the reaction terms are always set to $\sigma = \sigma _\textrm{S}= \sigma _\textrm{M}= 1$. In particular, we consider a diffusion-dominated case corresponding to $\nu = 1$, and a convection-dominated case corresponding to $\nu = 10^{-6}$. Finally, we set $\mu _{J_1}= 0.05$, $\mu _{J_2}= 0.01$ and $\mu _a = 10$ or $\mu _a = 20$ depending on $k=1$ or $k=2$. In Figs. 2 and 3, the numerical results are shown. We can observe that all the quantities converge at least with the expected order, see Corollary 21, and that the scheme is clearly robust with respect to the parameter $\nu$. In particular, the total error exhibits (at least) the expected $h^{1/2}$ gain in the pre-asymptotic reduction rate in the convection dominated case.

**Fig. 4:**
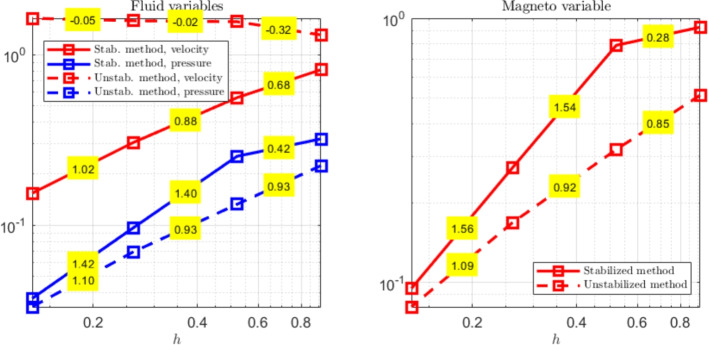
Numerical results for test 2, $k=1$

**Fig. 5:**
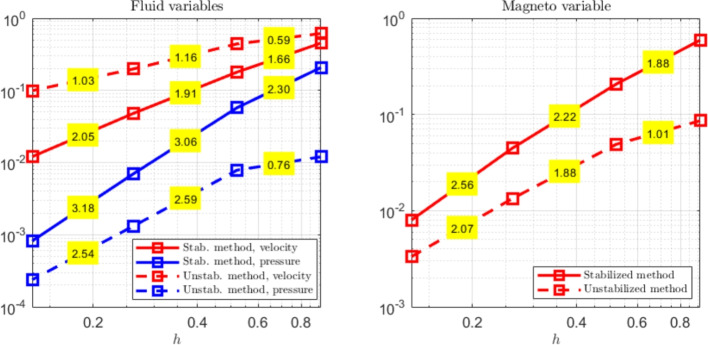
Numerical results for test 2, $k=2$

Test 2: Comparison with the unstabilized method. In this paragraph, we compare the stabilized method presented in this work with a more basic counterpart, obtained by removing the bilinear form $J_h(\cdot ,\cdot )$. Note that such version (later on labeled as “unstabilized”) still has some form of stabilization, given by the upwinding in the fluid convective term, but lacks a specific stabilization related to the magnetic field. We consider a realistic choice of the parameters, as in [6], by setting $\nu _\textrm{M}= 10^{-2}$ and $\nu _\textrm{S}= 10^{-10}$. In order to better underline the importance of the $J_h(\cdot ,\cdot )$ term, we purposely set $\boldsymbol{\chi }= 0$. Accordingly to [6], we impose $\mu _{J_1}= 5$ and $\mu _{J_2}= 0.01$. The remaining data, parameters and the analytic solution are chosen as in the previous numerical test. We consider the following error quantities: the $H^1-$seminorm of the velocity, the $H(\boldsymbol{\textrm{curl}})$-seminorm of the magnetic field, and the $L^2-$norm of the pressure. The numerical results for degree $k = 1$ are reported in Figure 4, while the results for $k = 2$ are reported in Figure 5. We observe that the unstabilized method struggles to achieve the optimal rate of convergence for the velocity field. In particular, for $k = 1$, the error in the velocity field remains constant. For the other error quantities, we observe that, during the first iterations, the unstabilized method provides a better approximation of the analytic solution; however, as the mesh is refined, the difference between the two methods becomes smaller. In particular, all the error slopes of the stabilized scheme are better than those of the unstabilized one.

Test 3: A benchmark on a non-convex domain. In this paragraph, we consider the non-convex domain depicted in Figure 1 (extruded L-shaped domain $\Omega = [-1,1]^3 \setminus ([-1,0)^2 \times [-1,1])$). The method proposed in [6] was devised for convex domains and, as such, makes use of $\boldsymbol{H}^1$-conforming discrete magnetic fields. Therefore, in particular, it requires the exact magnetic field $\textbf{B}$ to be in $\boldsymbol{H}^1(\Omega )$. We here develop a numerical benchmark in order to

1. check from the practical perspective this limitation of the scheme in [6];
2. show that the modified scheme here presented is suitable also for this kind of less regular problems.

Clearly, this comes at the price of a slightly higher dimensional system (when comparing the two schemes for the same order *k*).

We consider the solution of (9) given by the fields

$$ \boldsymbol{u}(x,y,z) := \begin{bmatrix} y^2 \\ z^2 \\ x^2 \end{bmatrix} \, , \qquad \boldsymbol{\chi }:= \begin{bmatrix} 1 \\ 2 \\ -1 \end{bmatrix} \, , \qquad \boldsymbol{\Theta }:= \begin{bmatrix} 1 \\ -1 \\ 2 \end{bmatrix} \, . $$

To construct the non-smooth solution for the magnetic field, we define the function

$$ r(x,y,z) = \sqrt{x^2+y^2}^\frac{2}{3} \sin \left( \frac{2}{3}\bigl (\arctan (y/x) + \frac{\pi }{2} \bigr )\right) \, , $$

and than we take

$$ \boldsymbol{B}(x,y,z) = \boldsymbol{\nabla }r(x,y,z) \, . $$

We note that since $\boldsymbol{B}$ is the gradient of a function, it holds $\boldsymbol{\textrm{curl}}(\boldsymbol{B}) = 0.$ Furthermore $\boldsymbol{B}\in [\boldsymbol{H}^{\frac{2}{3}}(\Omega )]^3$ but $\boldsymbol{B}\not \in [\boldsymbol{H}^{1}(\Omega )]^3$. We consider the same parameters of the previous example and we set $k=1$. For the pressure, we simply consider

$$ p(x,y,z) = 0 \, . $$

**Fig. 6:**
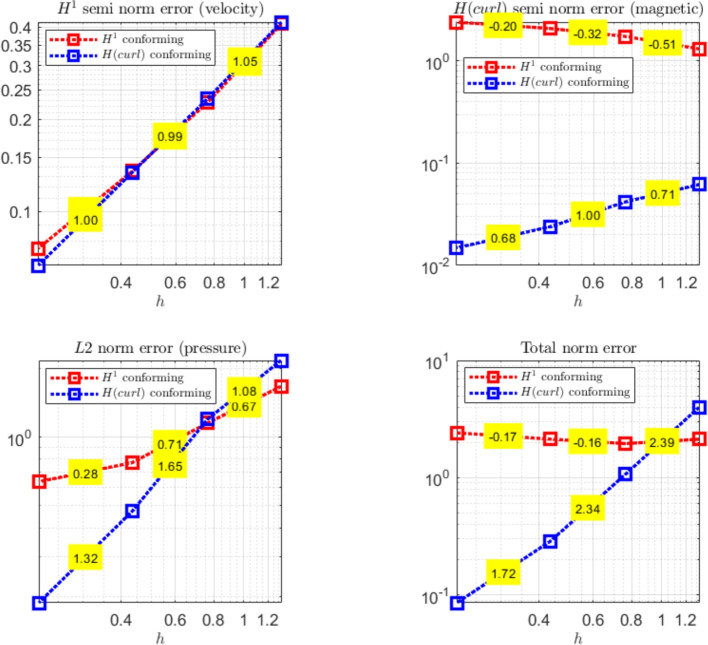
Numerical results for the third test case corresponding to $\nu = 1$

**Fig. 7:**
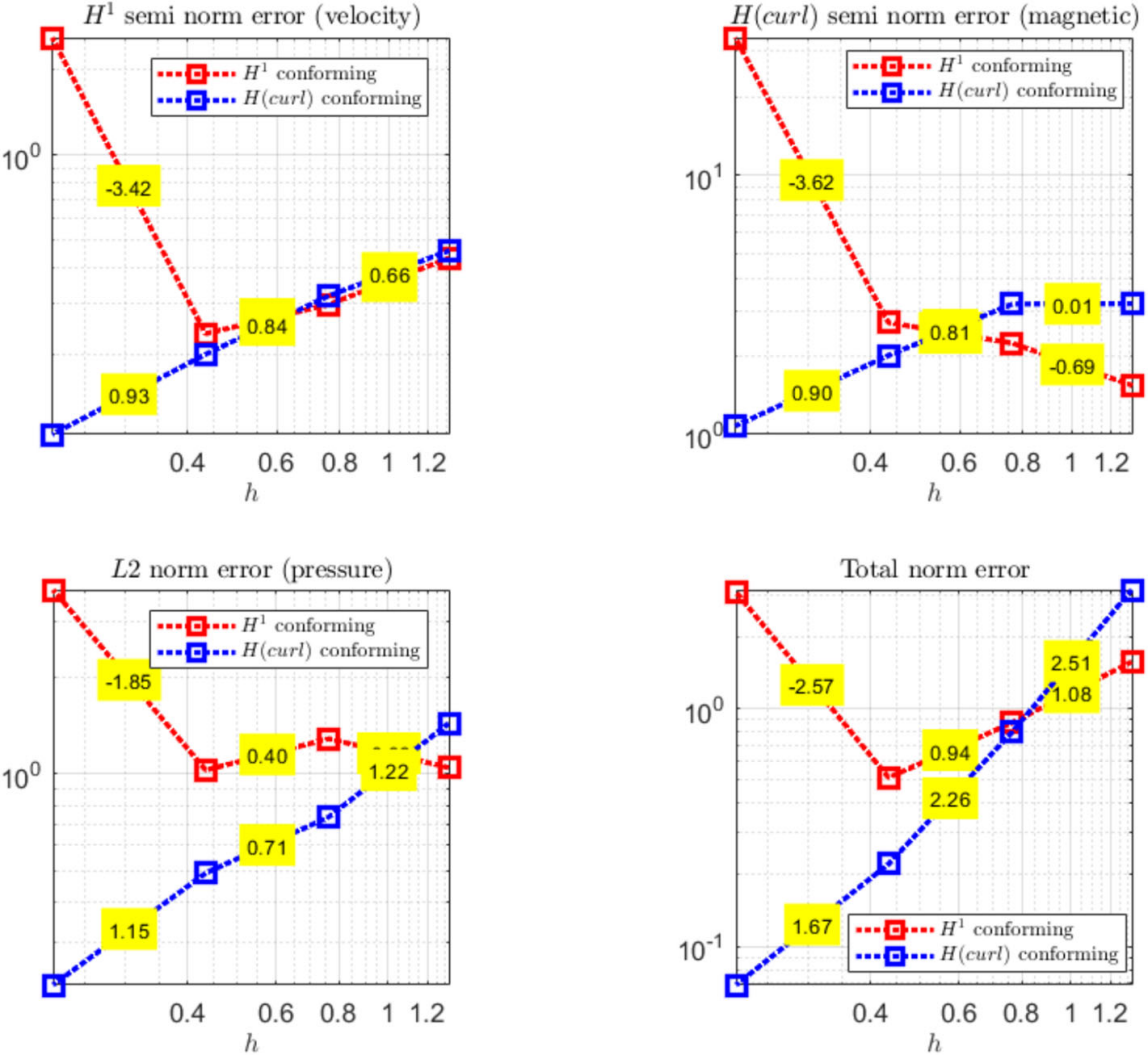
Numerical results for the third test case corresponding to $\nu = 10^{-6}.$

The results are shown in Figs. 6 and 7. We note that the approach proposed in [6] fails to reach the optimal rate of convergence in the non-convex domain, the results becoming even less reliable for small values of the parameter $\nu$. In fact, we observe that in many graphs the errors increase when reducing *h*. Contrary, our approach converge as expected.

Test 4: The Hartmann Flow. In this section, we consider the Hartmann channel flow problem [24]. This benchmark problem describes the flow of an electrically conducting fluid inside a channel, subjected to a constant transverse magnetic field. The computational domain is defined as $\Omega = [0,10] \times [-2,2] \times [-1,1]$. To align our method with the existing literature, in problem (13) we consider the Laplacian term $\Delta \boldsymbol{u}$ instead of the deformation tensor term $\boldsymbol{\textrm{div}}(\boldsymbol{\varepsilon }(\boldsymbol{u}) )$, with corresponding modifications in the definition of the bilinear form $a^\textrm{S}_h(\boldsymbol{u}_h, \boldsymbol{v}_h)$ in (14). The parameters are set to $\nu _\textrm{S}= \nu _\textrm{M}= 0.1$ and $\sigma _s = 0$, while $\sigma _m = 10^{-2}$ is chosen to be small but non-zero to ensure the stability of the method (without the need to enforce the solenoidal condition on $\boldsymbol{B}_h$ with a Lagrange multiplier). The convective field is set to $\boldsymbol{\chi }= 0$, while the linearized magnetic field is $\boldsymbol{\Theta }= (0,1,0)^T$. The jump parameters are set as in the previous experiments. For the magnetic equation, we impose Dirichlet boundary conditions given by

$$ \boldsymbol{B}\times \boldsymbol{n}= \begin{pmatrix} 0 \\ 1 \\ 0\end{pmatrix} \times \boldsymbol{n}$$

on the entire boundary $\partial \Omega$. Regarding the velocity, on the boundaries where $x = 0$ or $x = 10$, we impose Neumann boundary conditions given by

$$ p \boldsymbol{n}= p_N \boldsymbol{n}\, , $$

with $p_N = -10x + 100$. On the remainder of the boundary, we impose no-slip boundary conditions given by $\boldsymbol{u}= 0$. The order of the method is $k = 1$.

**Fig. 8:**
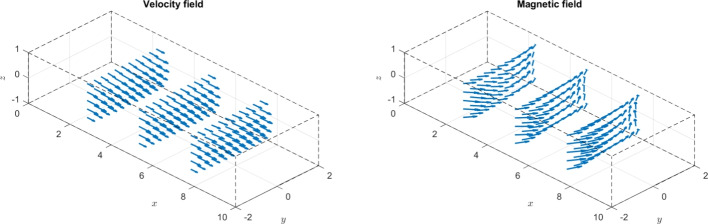
Velocity and Magnetic fields for the Hartmann flow problem

**Fig. 9:**
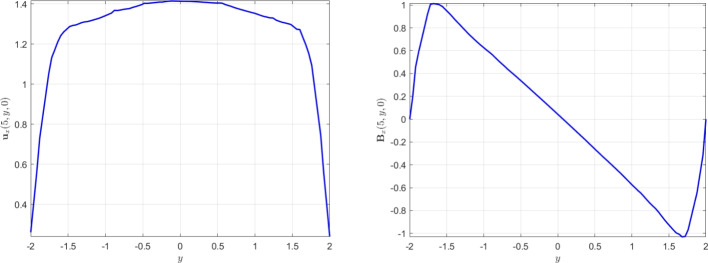
First component of the velocity and magnetic fields along $x=5$, $z = 0$ and $-2 \le y \le 2.$

We consider a decomposition of the domain made by 40150 tetrahedrons. The numerical results are reported in Figs. 8 and  9. We observe the characteristic flattening of the velocity profile in the core region of the channel. Near the walls $y = \pm 2$, the formation of the boundary layers is clearly visible. Regarding the magnetic field, we observe two distinct components. The first one, directed along the *y*-direction, corresponds to the imposed field, while the induced component along the *x*-direction exhibits the typical antisymmetric profile.
